# 2-Selenouridine, a Modified Nucleoside of Bacterial tRNAs, Its Reactivity in the Presence of Oxidizing and Reducing Reagents

**DOI:** 10.3390/ijms23147973

**Published:** 2022-07-19

**Authors:** Katarzyna Kulik, Klaudia Sadowska, Ewelina Wielgus, Barbara Pacholczyk-Sienicka, Elzbieta Sochacka, Barbara Nawrot

**Affiliations:** 1 Centre of Molecular and Macromolecular Studies, Polish Academy of Sciences, Sienkiewicza 112, 90-363 Lodz, Poland; ms@cbmm.lodz.pl (E.W.); bnawrot@cbmm.lodz.pl (B.N.); 2 Institute of Organic Chemistry, Lodz University of Technology, Zeromskiego 116, 90-924 Lodz, Poland; klaudia.sadowska@dokt.p.lodz.pl (K.S.); barbara.pacholczyk@p.lodz.pl (B.P.-S.); elzbieta.sochacka@p.lodz.pl (E.S.)

**Keywords:** 2-selenouridine, wobble nucleoside, diselenide, oxidative stress, tRNA

## Abstract

The 5-substituted 2-selenouridines are natural components of the bacterial tRNA epitranscriptome. Because selenium-containing biomolecules are redox-active entities, the oxidation susceptibility of 2-selenouridine (Se2U) was studied in the presence of hydrogen peroxide under various conditions and compared with previously reported data for 2-thiouridine (S2U). It was found that Se2U is more susceptible to oxidation and converted in the first step to the corresponding diselenide (Se2U)_2_, an unstable intermediate that decomposes to uridine and selenium. The reversibility of the oxidized state of Se2U was demonstrated by the efficient reduction of (Se2U)_2_ to Se2U in the presence of common reducing agents. Thus, the 2-selenouridine component of tRNA may have antioxidant potential in cells because of its ability to react with both cellular ROS components and reducing agents. Interestingly, in the course of the reactions studied, we found that (Se2U)_2_ reacts with Se2U to form new ‘oligomeric nucleosides′ as linear and cyclic byproducts.

## 1. Introduction

**Figure 1:**
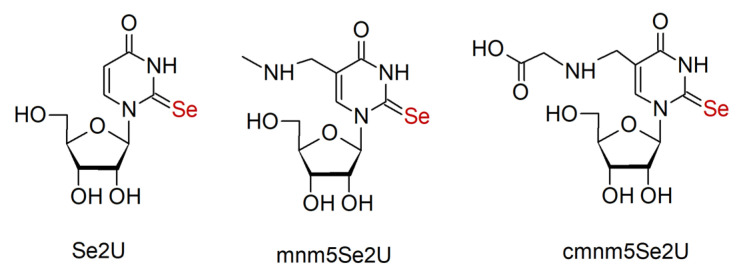
The structures of 2-selenouridine Se2U and its 5-substituted counterparts mnmSe2U and cmnmSe2U present in wobble position of bacterial tRNAs.

Transfer RNAs are adapter molecules in the translational machinery that supply amino acid residues to the growing peptide chain, according to the genetic information encoded by the messenger RNA (mRNA). The readout of mRNA codons by tRNA anticodons is a dynamic process controlled by cellular conditions and the structure of the interacting molecules. Post-transcriptional modifications of mRNA and tRNA (as well as other non-coding and regulatory RNAs) are the major functional players of the cellular epitranscriptome [1,2,3,4,5]. In this context, 5-substituted uridines and 2-thiouridines at the first position of tRNA anticodons (position 34, the so-called ‘wobble′ position) play a key role in the ability of tRNA anticodons to accurately recognize their cognate mRNA codons [6,7,8]. These two groups of modifications are present in anticodons of the tRNA *iso*-acceptors specific for lysine, glutamate, and glutamine in all three domains of life. While the function of sulfur-modified uridines containing various R5 substituents of the nucleobase ring has been extensively studied, in terms of their cellular function and influence on the translation process [9,10,11,12,13,14], less is known about the biological function of 5-substituted 2-selenouridines (Se2U, **1**), which has been so far univocally identified in bacterial systems. To date, the abundant 5-methylaminomethyl-2-selenouridine (mnm5Se2U) has been found in tRNA epitranscriptomes of bacterial origin in tRNA^Lys^ and tRNA^Glu^, and 5-carboxymethylaminomethyl-2-selenouridine (cmnm5Se2U) has been found in tRNA^Gln^ (Figure 1) [15,16,17,18,19].

The 2-selenouridines in the tRNA chain are synthesized from their 2-thiouridine precursors, but the mechanism of this conversion has been elusive [18,20,21]. We recently found that the enzymatic conversion of S2U-RNA to Se2U-RNA occurs in two steps, namely by (i) geranylation of the S2U residue at the sulfur atom (geS2U-RNA) and (ii) displacement of the thio-geranyl residue by endogenous selenophosphate, while the resulting phosphorylated 2-selenouridine RNA is hydrolyzed to the final selenium derivative (Se2U-RNA) [22,23,24]. Both processes are catalyzed by tRNA 2-selenouridine synthase (SelU), a two-faced enzyme whose structure is not yet known, suggesting a plausible mechanism for the conversion of S2U-tRNA to Se2U-tRNA in the cell.

However, why nature evolved this novel enzymatic process and introduced selenium into the wobble uridines of tRNA is not yet known. The recent literature reports on the physicochemical properties of sulfur- and selenium-containing uridines suggest that R5Se2U34, similar to R5S2U34, may play a role in regulating the translation process by modulating codon-anticodon recognition and reading off the synonymous codons NNG-3′ and NNA-3′ within mRNA [25,26]. This is a particularly interesting problem in the bacterial system, where a single tRNA^Lys^ reads both AAA -3′ and AAG-3′ codons, while a single tRNA^Glu^ accepts the GAA-3′ and GAG -3′ codons [8,12,27,28]. Biological experiments have shown that, indeed, both synonymous codons can be recognized by R5S2U-tRNAs, with some preference for the A3′-ending codons. On the other hand, in vitro experiments using a wheat germ extract-rabbit globin mRNA translation system have shown that G3′-ending codons for Lys and Glu in globin mRNA are preferentially recognized by bacterial aminoacylated Se2U-tRNAs when their 2-thio *iso*-acceptors are compared [29].

In answering the above question, we turned to the chemistry of proteins in which amino acids modified with selenium, such as L-selenocysteine (Sec) and L-selenomethionine, which are essential for the activity of enzymes found in all kingdoms of life [30,31,32]. L-selenocysteine has antioxidant activity resulting from its ability to reduce reactive oxygen species. It plays a role in the formation and recycling of glutathione, an important endogenous antioxidant in many organisms, including humans [33,34,35]. Considering this aspect, our research in recent years has focused on the susceptibility of 2-thiouridine (S2U) to oxidative stress. We have previously shown that S2U, either as a free nucleoside or incorporated into the RNA chain, is irreversibly desulfurized to uridine in the presence of an excess of hydrogen peroxide or stronger oxidants, such as potassium peroxymonosulfate (Oxone^®^), via sulfenic (U-SOH), sulfinic (U-SO_2_H), and sulfonic (U-SO_3_H) intermediates to release sulfuric acid (IV or VI) or via a carbene intermediate to 4-pyrimidinone ribonucleoside (H2U) [36,37]. The ratio of the final products U and H2U depends on the pH, concentration of the reagents used, and R substituents at position 5 of the nucleobase [38,39]. As shown by the hybridization experiments, the replacement of S2U by H2U in the RNA chain leads to a loss of adenosine readability (due to the alteration of the hydrogen bond donor and acceptor pattern), which may affect tRNA functionality under cellular conditions [11].

Selenium-modified nucleosides are of great therapeutic interest because of their potential as antiviral and anticancer agents. One of the various selenium modifications are nucleosides with a selenium atom in the sugar ring, as third generation nucleosides developed to overcome some of the bioavailability limitations of 4-oxo, 4-thio, and 4-carbanucleosides [40,41,42,43]. Moreover, the organoselenium compounds were designed and evaluated for their antimicrobial activity, given the promising biological activities against some bacteria and fungi. In in vitro and in vivo studies, selenium containing organic compounds have demonstrated their activity against drug resistant bacteria and their potential as an effective therapeutic agent in acute wound infections [44,45,46,47].

To date, there are no data on the accessibility of 2-selenouridine to oxidants, except for two studies performed with 2-selenonucleobases, i.e., 5-carboxy-2-selenouracil (non-natural c5Se2Ura) and 5-non-substituted 2-selenouracil (Se2Ura), the component of natural 2-selenouridine present in transfer RNAs [32,48]. Although the general conclusions from these data suggest a reversible oxidation mechanism of selenium-containing nucleobases under reducing conditions, the conversion pathways to stable oxidation products of c5Se2Ura and Se2Ura vary considerably and are not consistent in any of the reports.

Here, we performed the studies with the aim to elucidate, in detail, the process of oxidation of 2-selenouridine (**1**, Figure 1) under various conditions that mimic oxidative stress in the cell and propose the mechanism of Se2U oxidation. Considering the redox properties of selenium compounds, which are quite different from their sulfur analogs, we were interested in identifying individual intermediates and final products of 2-selenouridine oxidation and comparing them with known products of 2-thiouridine oxidation [36,37,38,39]. The course of hydrogen peroxide-assisted Se2U oxidation was studied as a function of the buffer pH, reactants concentration, and temperature of the process. To assess the reversibility of the oxidized state of Se2U, the reactions were tested in the presence of common reducing agents, such as ascorbic acid, dithiothreitol, and glutathione.

## 2. Results

### 2.1. Analysis of the Oxidation Course of Se2U (**1**)

**Scheme 1:**
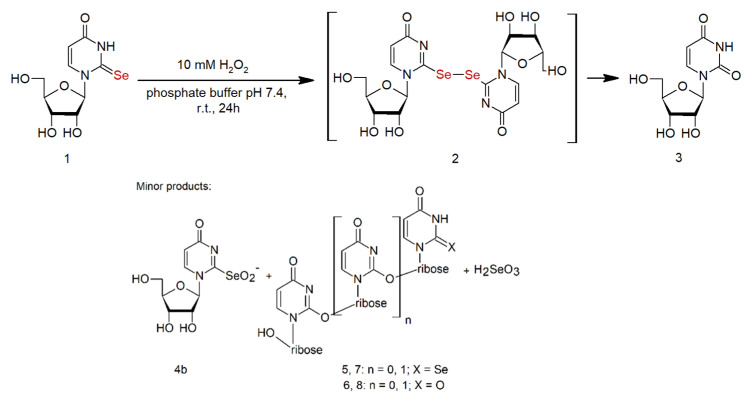
Oxidation products of 2-selenouridine (**1**).

**Figure 2:**
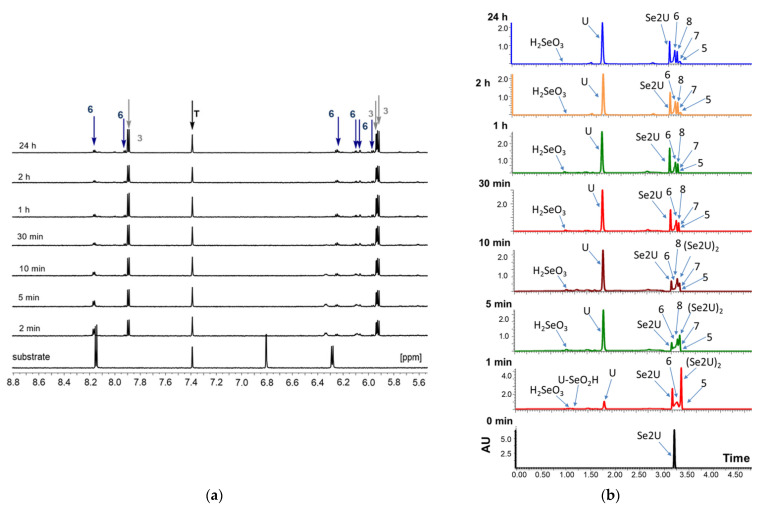
(**a**) ^1^H NMR analysis of the reaction mixtures for the oxidation of Se2U (**1**, 10 mM) with H_2_O_2_ (10 mM) in 67 mM phosphate buffer at pH 7.4 and room temperature at the indicated time point. The chemical shifts (δ, ppm) for protons H5, H6, and H1′, as well as the coupling constants *J_H6-H5_* and *J_H1′-H2′_*, as well as the *m*/*z* values for all identified compounds are given in Table 2. A total of 5 mM thymine was used as an internal standard; (**b**) UPLC–PDA chromatographic analysis of the time course of products formation for the oxidation reaction of Se2U (**1**, 10 mM) with H_2_O_2_ (10 mM) in 67 mM phosphate buffer at pH 7.4 and rt. The *m*/*z* values are given in Table 2. The inorganic selenous acid was identified by UPLC-ESI(-)-HRMS, and its retention time was determined based on the extracted ion chromatograms (EICs) for the ion corresponding to the deprotonated molecule (*m*/*z* 128.909).

To identify possible products of the oxidation reaction of Se2U (**1**), the reaction was carried out with H_2_O_2_ at a molar ratio of 1:1 in phosphate buffer pH 7.4 (Scheme 1 and Experimental Section, Experiment #1, Table 1, where all experiments were summarized). During the first phase of this reaction, dynamic changes in the composition of the reactants were observed by spectral analysis (Figure 2a,b). The ^1^H NMR resonance signals corresponding to **1** (δ 8.14, 6.83, and 6.28 ppm; (*m*/*z* 306.9834) disappeared completely after only two minutes, whereas small broad signals assigned to diselenide **2** appeared at the chemical shifts of δ 8.16, 6.11, and 6.34 ppm (*m*/*z* 612.9600). Diselenide **2** proved to be a very reactive compound, as it rapidly decomposed to uridine **3** (δ 7.78, 5.82, and 5.80 ppm, as well as *m*/*z* 243.0623), with a yield of 71%. These changes were accompanied by a gradual release of a red precipitate, indicating deselenation of **2** by elimination of selenium. The electron impact (EI) mass spectrum (Figure S24) recorded for the separated precipitate confirmed that it was elemental selenium (Se^0^), although it was not possible to quantify the content of this product in the reaction mixture. The progress of oxidation reaction **1** in the presence of a 10-fold excess of H_2_O_2_ (experiment #5) was much faster, as no signals were observed for intermediate **2**, and uridine was obtained as the only organic product of this transformation (Figures S29 and S30). Reaction #1 gave several minor nucleoside products, **5**–**8**, the structure of which will be discussed later, as well as an acidic product of U-SeO_2_H (**4b**) and inorganic selenous acid (H_2_SeO_3_). Importantly, the use of hydrogen peroxide to 2-selenouridine in a 1:1 molar ratio was sufficient to convert most of the substrate **1** to diselenide **2**.

### 2.2. Influence of Medium pH on the Course of the Se2U Oxidation

**Figure 3:**
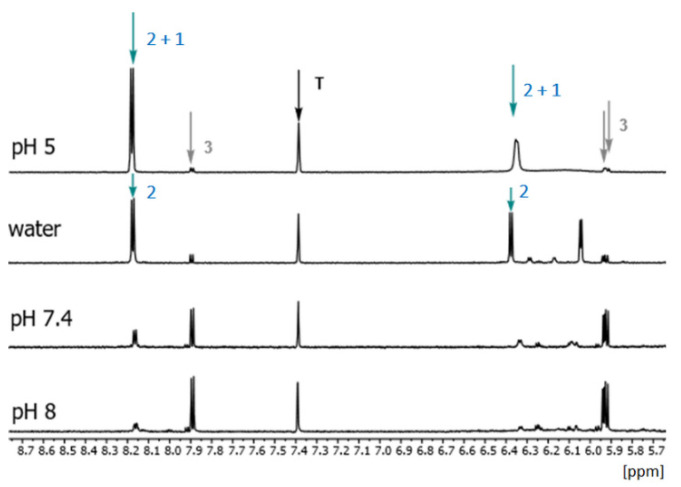
pH-dependent uridine formation in the reaction of 10 mM Se2U with 10 mM H_2_O_2_ at room temperature and in phosphate buffers pH 8.0, 7.4, and 5.0, as well as in deionized water (pH 6.5), observed by ^1^H NMR monitoring 2 min after the start of the reaction. The arrows indicate the resonance signals for **2**, **3**, and the reference thymine (T) used at the same concentration (5 mM).

The effect of pH on the course of the oxidation reactions of **1** (reactions #1–4) was investigated by determining the content of uridine (**3**) in the reaction mixture after 2 min of H_2_O_2_ addition. As shown in Figure 3 and Figures S31–S33, the reaction performed in phosphate buffer pH 8.0 gave the highest content of **3** (45%) after 2 min, but the final yield of uridine was much lower (53%) than for the reaction performed at pH 5 (5% after 2 min), which, finally (after 24 h), resulted in an almost quantitative yield of **3** (Table 3). Thus, the rate of formation of diselenide and its decomposition to the main product **3,** and in addition to other products (compounds **5**–**8**), increased at higher pH. The reactions carried out under acidic conditions (in deionized water with pH 6.5 and phosphate buffer pH 5.0) were slower, since diselenide **2** was still the main product under these conditions. However, accurate determination of the content of **2** was impossible because dynamic exchange process between **1** and **2** occurring simultaneously in the reaction mixture, was clearly visible (we will discuss this in a subsequent section). This is illustrated by a broad signal at δ, about 6.30 ppm in the spectrum at pH 5, which is the average signal of the H1′ anomeric protons of **1** and **2**.

### 2.3. Products with the Structure of Selenium Oxoacids in the Oxidation of Se2U

Observation of the ^1^H NMR spectra of the reactions carried out in water in molar ratios of 1:1 and 1:10 (reactions #4 and #6, respectively), from 2 to 120 min, indicates the formation of some amounts of two intermediates whose resonance signals at δ 8.18 (H6), 6.31 (H5), and 6.19 (H1′), as well as at δ 8.13 (H6), 6.26 (H5), and 5.86 ppm (H1′), respectively, are found (Figures S33 and S34). Parallel UPLC-HRMS analysis showed that the first of them is most likely a seleninic acid derivative **4b** (U-SeO_2_H, *m*/*z* 338.9720) (Figures S35 and S36). Although the latter was not identified in the bulk analysis, it seems most likely to be the selenenic acid derivative **4a** (U-SeOH), the compound that probably could not be detected under the conditions of LC–MS because of its high reactivity. The seleninic acid derivative of uridine U-SeO_2_H, similarly to the sulfinic acid derivative of uridine U-SO_2_H, could be substituted by a water molecule at the C2 atom of the nucleoside, thus leading to the elimination of selenium oxide (IV) [37,39]. The presence of the inorganic selenious acid H_2_SeO_3_ (*m*/*z* 128.909), over the course of up to 24 h, supports this hypothesis and suggests that it is one of the products of the **1** deselenation pathway, although not the dominant one, in contrast to the chemistry of oxidation of 2-thiouridine [37]. It is worth noting that, in the oxidation reactions carried out in deionized water (reactions #4 and #6), in which acidic products are released, the pH of the deionized water in the reaction course decreased from 6.5 to a lower pH (in the case of an analogous process carried out on Se2Ura the pH drops to 2.5) [48]. Thus, lowering the pH of the reaction solution indicates the appearance of some amounts of acidic reaction products **4**, i.e., selenenic acid U-SeOH (**4a**) and seleninic acid U-SeO_2_H (**4b**), indicating that the small portion of **1** is converted to uridine via oxidized forms of selenium. However, selenonic acid U-SeO_3_H was not identified under the conditions of the reactions studied, in contrast to S2U oxidation, where the sulfonic acid U-SO_3_H counterpart was observed [37].

### 2.4. The Chemical Exchange between Se2U and (Se2U)_2_ in ^1^H NMR Time Scale

**Figure 4:**
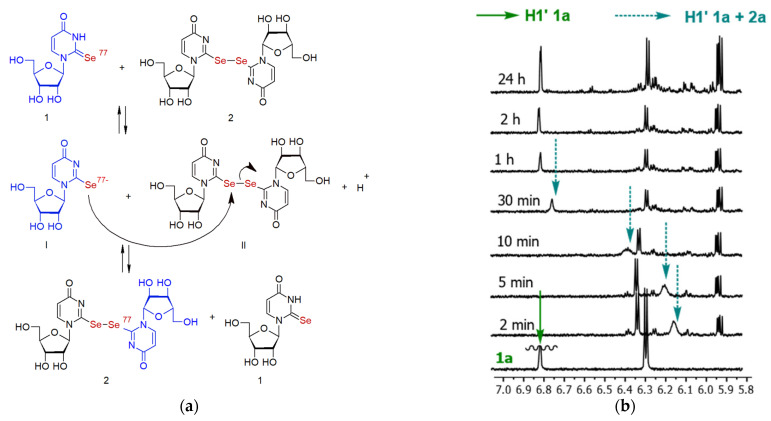
(**a**) Schematic presentation of the Se2U/(Se2U)_2_ exchange reactions performed with diselenide (Se2U)_2_ (containing a selenium isotope mixture of natural abundance) and ^77^Se2U (containing 100% ^77^Se), as discussed in Sect. 2.6; (**b**) ^1^H NMR spectral set collected for the oxidation Se2U with a molar ratio of **1** to H_2_O_2_ of 1:0.5, observed between 2 min and 24 h of the reaction course. The signals of H1′ of **1** at δ 6.80 ppm and **1**/**2** exchange species (at δ 6.15 ppm/2 min, 6.20/5 min, 6.4/10 min, and 6.75 at 30 min) are indicated by the arrows.

In the chemistry of selenol (R^1^SeH) and diselenide derivatives (R^2^Se)_2_, the process of the exchange of the R^1^ and R^2^ groups (R^1^↔R^2^) is well-documented by NMR spectroscopy in aqueous or organic solutions. It can be observed for the mixtures of both derivatives when it proceeds in the ^1^H or ^77^Se NMR time scale. Recently, Landry has reported such an exchange of R^1^ and R^2^ groups between two stable compounds, R^1^SeH and (R^2^Se)_2_, for which the chemical shifts in the ^77^Se NMR spectra are different [49]. However, solutions consisting of a mixture of these two compounds shows a single set of NMR resonances appearing in the weighted average of the signals from the pure components. This process depends on the deprotonation of selenol R^1^SeH to selenolate R^1^Se^−^, which is thought to be the species responsible for the effective exchange of the R^1^ group for the R^2^ group in (R^2^Se)_2_. On this basis, a reasonable mechanism was proposed involving (i) the transfer of protons from R^1^SeH to one of the nitrogen atoms of (R^2^Se)_2_ to generate R^1^Se^−^ and (R^2^Se)_2_H^+^, followed by (ii) a nucleophilic attack of R^1^Se^−^ on (R^2^Se)_2_H^+^ to release R^2^SeH and form R^1^SeSeR^2^ (Figure 4a).

To answer the question of whether such an exchange process is observed for the selone Se2U and diselenide (Se2U)_2_ studied here, we had to use a mixture in which both compounds are present for at least a limited time. As shown earlier, diselenide **2** is the very unstable derivative under the oxidation conditions, as it is decomposed to uridine and selenium. Our attempts to isolate the diselenide **2** entity from the oxidation reaction mixture (RP-HPLC) and characterize it were unsuccessful. Therefore, we decided to spectrally monitor the progress of the oxidation reaction carried out with a limited amount of hydrogen peroxide over Se2U (molar ratio 1:0.5) at room temperature and phosphate buffer pH 7.4 (reaction #8). Under these conditions, we assumed that we could observe the exchange reaction in the ^1^H NMR time scale between the substrate Se2U and intermediate (Se2U)_2_ early in the oxidation process, as long as the diselenide was present in the reaction mixture. Although, in this case, the ligands were the same (uridine selenolate anion), we should still observe an altered ^1^H NMR spectrum; indeed, in the spectrum recorded 2 min after the start of the reaction, the H1′ signal for Se2U (6.80 ppm) disappeared, while a new broad signal of 6.15 ppm appeared (Figure 4b). As time progressed (5, 10, and 30 min), this signal shifted to a lower field (higher chemical shift values) and, finally, reached the chemical shift of 6.80 ppm of pure Se2U (after about 1 h). This picture of the course of the reaction results from the decomposition of diselenide **2** (mainly to **3**) and, consequently, the reduction of its amount at a constant amount of **1** in the reaction mixture.

Selenol/diselenide exchange reactions were evidenced by the fact that, under the conditions used, i.e., in phosphate buffer with a pH of 7.4, about half of the selone **1** was deionized (p*K*_a_ 7.30 [26]) and formed the selenolate anion I (Figure 4a). In the next step, the positively charged diselenide II reacted with selenolate I, forming the neutral form of diselenide **2** by exchange process (equilibrium reactions). Interestingly, the same process performed at a lower temperature (10 °C, reaction #9) showed a slower shift of the averaged signal toward the lower field (Figure S37), which may be a result of the altered kinetics of the processes that eventually lead to exchange between selenol and diselenide, compared with the process performed at room temperature (Figure 4b, reaction #8).

### 2.5. Monitoring of the Progress of Se2U Oxidation by ^77^Se NMR

**Figure 5:**
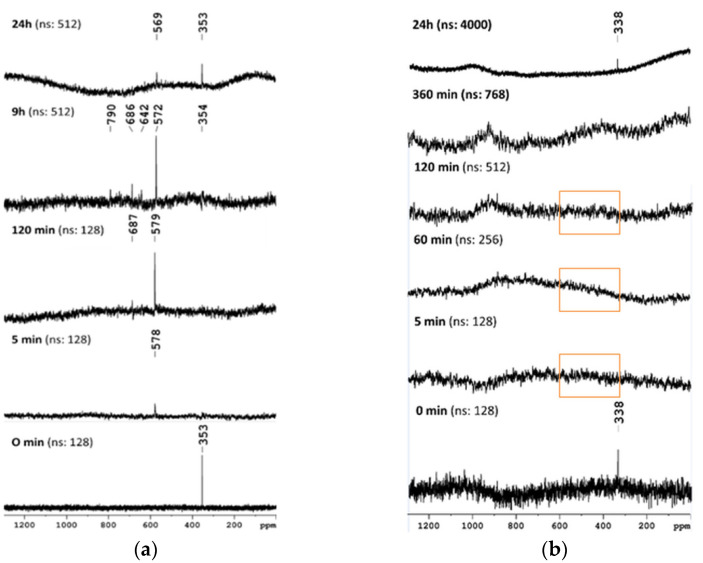
(**a**) Time-dependent ^77^Se NMR analysis of the reaction mixtures for the oxidation of Se2U (**1**, 40 mM) with H_2_O_2_ (20 mM) in water and (**b**) phosphate buffer pH 7.4 at 10 °C; ^77^Se chemical shifts are marked, and the number of scans is indicated for each spectrum.

The selenium-containing substrate used in the synthesis of **1**, which was required for ^77^Se NMR analysis, contained an isotopically pure ^77^Se isotope, in contrast to the ‘common′ Se2U, with a ^77^Se isotope abundance about 7.6%. Our previous studies have shown that the kinetics of the Se2U oxidation process is pH-dependent and slower in acidic pH. Therefore, the course of oxidation of Se2U (**1**, 40 mM) (the 99.6% abundance of ^77^Se present in **1** was documented by an extended ESI mass spectrum, Figure S3) in the presence of H_2_O_2_ (20 mM), which was studied by ^77^Se NMR in water at a temperature of 10 °C (Figure 5a, experiment #12). The resonance signal of δ 353 ppm in the spectrum of substrate (**1**) was attributed to the selone form (C=Se) of Se2U, according to the literature reports [50,51,52]. The addition of 0.5 equiv. H_2_O_2_ resulted in a rapid conversion of **1** to the diselenide **2**, showing a ^77^Se signal at δ 579 ppm. After about 2.5 h, weak resonance signals appeared in the spectrum at δ 642 and 686 ppm, possibly corresponding to products that could not be detected under the LC–MS conditions because of its high reactivity. During the course of the reaction, **2** decomposed to U and minor organic products, so that a gradual decrease in the intensity of the signal corresponding to the diselenide **2** was observed. After 24 h, only two weak signals were observed, as detected with a large number of scans, representing the remaining amount of substrate **1** (at δ 353 ppm) and minute amount of diselenide **2** (at δ 569 ppm). The used conditions (lower pH, 10 °C) did not allow for exchange reactions between selenol/diselenide to be observed, either by ^77^Se NMR or ^1^H NMR (data not shown).

However, for the reaction performed at the same temperature (10 °C) but higher pH (pH 7.4, reaction #13), the ^1^H NMR analysis confirmed the exchange process between Se2U and (Se2U)_2_ (Figure S39), while the simultaneously registered ^77^Se NMR spectrum showed no signal (region marked by orange boxes), neither for the individual components nor the averaged signal between the expected range of signals characteristic of **1** and **2** (Figure 5b). We assumed that the disappearance of the mean signal was due to the broadening of the ^77^Se resonance line, and the reduction of the signal-to-noise ratio was due to the exchange reactions between selenol/diselenide. Interestingly, at pH 7.4 the ^77^Se resonance signal of the substrate Se2U shifted towards a higher field (to δ 338 ppm) and was much noisier than in the spectrum of the reaction carried out in water, despite the fac that the Se2U and H_2_O_2_ concentrations were the same. The observed chemical shift and line broadening were probably due to the deprotonation of selenol Se2U to selenolate form at pH 7.4, as its p*K*_a_ was 7.30 and ca. was 50% in ionized form, in contrast to more acidic conditions [26,53,54]. Moreover, we can conclude that the exchange process between selenol/diselenide depends more on the buffer pH than on the temperature, as shown by the ^77^Se NMR studies in water and at pH 7.4. Although the observed phenomenon has little, if anything, to do with cellular processes, it is interesting to learn about the properties of 2-selenouridine, a natural component of transfer RNAs.

### 2.6. The Chemical Exchange between ^77^Se2U and (Se2U)_2_ Monitored by LC–MS

**Figure 6:**
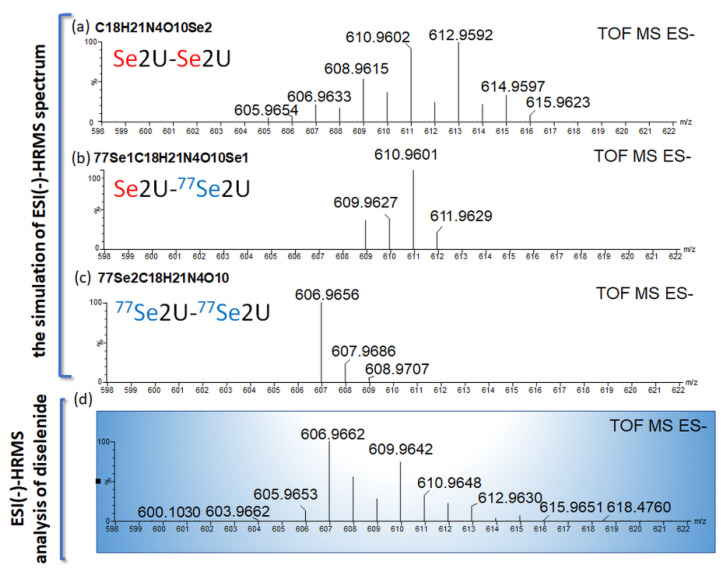
The LC–MS exchange experiment was performed with diselenide (Se2U)_2_ (containing a selenium isotope mixture of natural abundance) and ^77^Se2U (containing 100% ^77^Se). The reaction was carried out in water at rt. for 5 min, and then the spectrum ((**d**), in blue rectangle) was recorded. It shows the signals for diselenide ^77^Se-^77^Se, diselenide ^77^Se-Se, and Se-Se with characteristic selenium isotope distribution. Spectra (**a**–**c**) represent simulated spectra for respective, differently labeled diselenides.

Finally, we attempted to demonstrate the exchange process between two isotopically different selenium-containing uridine derivatives (7.6% and 99.6% selenium-77 abundance). Thus, (Se2U)_2_ was carefully isolated from reaction #8 (RP-HPLC) and, after partial removal of the solvent, immediately mixed with ^77^Se2U (one equivalent of the amount of Se2U used for the synthesis of diselenide), and the mass spectrum of this reaction mixture was recorded after 5 min (Figure 6d). For comparison, the simulated spectra for each of the mixture components are shown in Figure 6a–c. Both compounds, ^77^Se2U and (Se2U)_2_, react and form a mixture of ^77^SeU-Se2U, ^77^SeU-^77^SeU, and Se2U-Se2U. This result is definitive evidence for the exchange reactions between 2-selenouridine and its diselenide.

### 2.7. Oligomeric Side Products of the Condensation of Diselenide **2**

**Figure 7:**
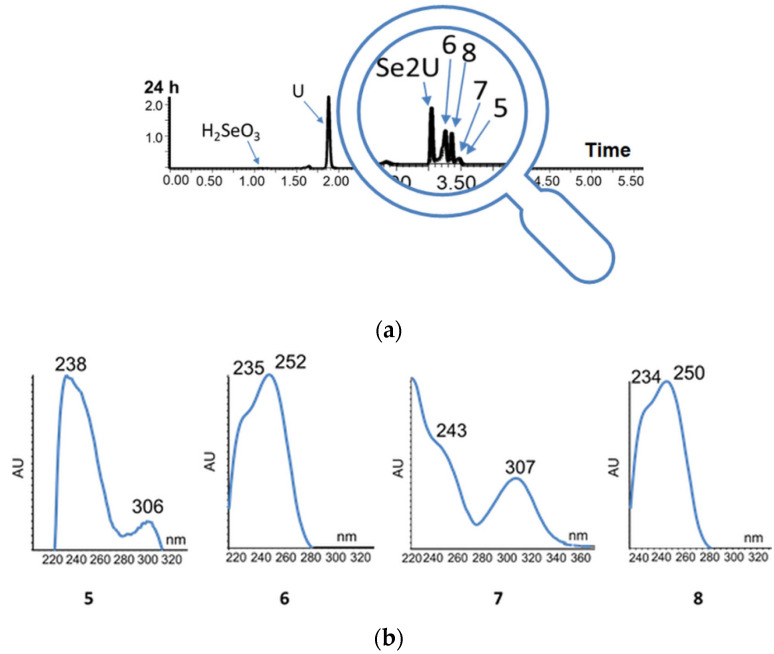
(**a**) UPLC–PDA chromatographic analysis of the oxidation reaction of Se2U (**1**, 10 mM) with H_2_O_2_ (10 mM) in 50 mM phosphate buffer at pH 7.4 and rt. (#1); (**b**) absorption spectra of dinucleosides **5**, **6** and trinucleosides **7**, **8**.

Small amounts of organic side products were observed in the ^1^H NMR spectrum of reaction #1 after 24 h, corresponding in mass spectrometric analysis to compounds **5** (*m*/*z* 533.0418) and **7** (*m*/*z* 759.102) and containing a selenium atom, as well as compounds **6** (*m*/*z* 469.1206, yield about 8%) and **8** (*m*/*z* 695.1798). The *m*/*z* values of the ions of compounds **6** and **8**, referring to their deprotonated forms, were 63.9222 amu smaller than those of products **5** and **7**, indicating that, in these compounds, the selenium atom has been replaced by an oxygen atom. These were new, unknown compounds, for which careful UPLC–ESI(-)-MS analysis revealed that compounds **5** and **6** were dinucleosides and compounds **7** and **8** were trinucleosides. The fragment of the UPLC–PDA chromatogram of the oxidation reaction of Se2U (reaction #1) is shown in Figure 7a. It shows clear signals for compounds **6** and **8** and less intense peaks for compounds **7** and **5**. In addition, analysis of UV–VIS spectrum (Figure 7b) indicated that the compounds contain chromophores that absorb at a maximum of about λ 234–243 nm (4-pyrimidinone riboside scaffold [55]) and λ 250–252 nm (uridine scaffold) for **6** and **8** or λ 306–307 nm (2-selenouridine scaffold [24]) for **5** and **7**.

**Figure 8:**
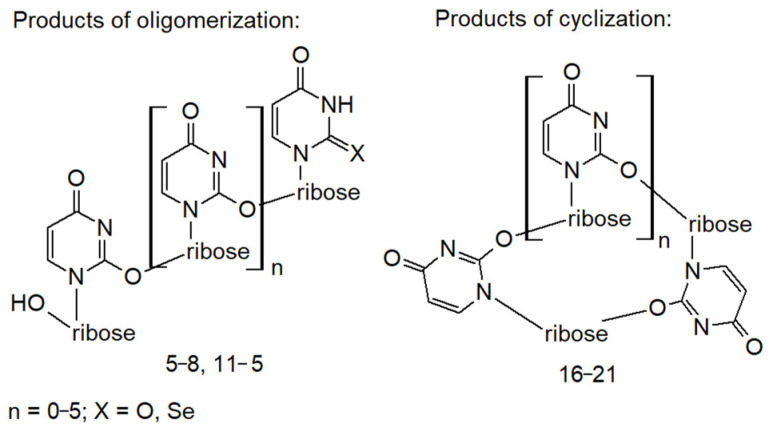
Structure of the oligomerization products **5**–**8** and **11**–**15** and their cyclized forms **16**–**21**.

**Figure 9:**
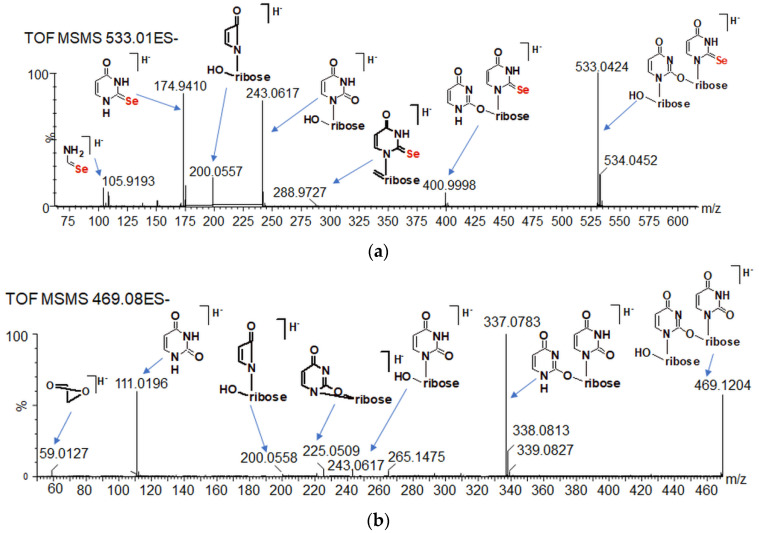

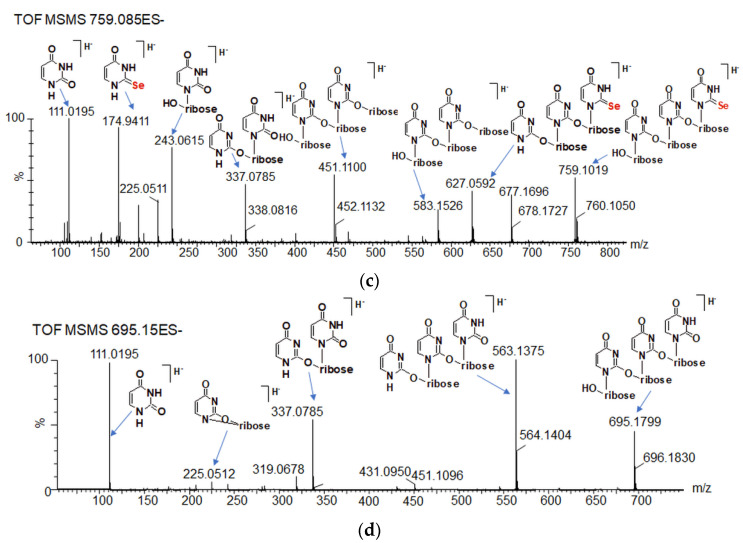
UPLC/ESI(-)-MS/MS spectra of the [M-H]^−^ of **5** (**a**), **6** (**b**), **7** (**c**), and **8** (**d**) recorded with collision energy ramping from 15 to 35 eV.

Interestingly, detailed UPLC–ESI(-)-MS of the reaction mixture also allowed for the identification (besides **5**–**8**) of a minute amount of several other oligomers, as shown in Table 4, including linear compounds composed of 4 to 7 nucleosides in length (**11**–**15**) and corresponding macrocyclic analogs (**16**–**21**, Figure 8). Covalent bonding between two nucleosides was confirmed by the presence of fragmentation ions at *m*/*z* 400.9998 for **5** and *m*/*z* 337.0783 for **6** in the collision-induced dissociation spectra (CID, Figure 9a,b). In contrast, the presence of trinucleoside compounds was confirmed by fragmentation ions at *m*/*z* 627.0592 and 583.1526 for **7** and *m*/*z* 563.1375 for **8** (Figure 9c,d).

**Figure 10:**
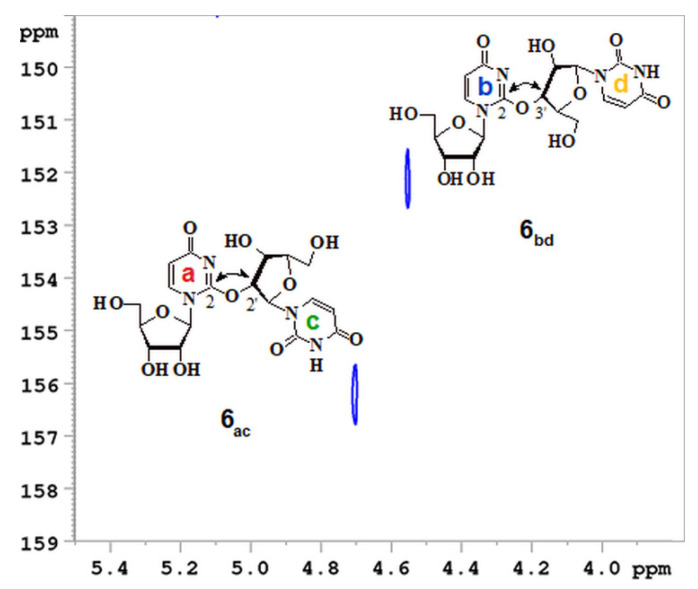
HMBC spectrum of **6** (regioisomer C2-O2′ with nucleobase rings ac and 2C-O3′ with nucleobase rings bd) and an extended region of the HMBC spectrum (^1^H: 4.0–5.4 ppm and ^13^C: 149–159 ppm) (D_2_O).

Separation of pure oligomeric compounds with similar chromatographic properties from a complex mixture of oxidation products was a challenging purification problem. However, we succeeded in isolating minute amounts of dinucleoside **6**, which had a well-defined molecular mass in the UPLC–ESI(-)-HRMS analysis. However, the ^1^H NMR spectrum showed that it is a mixture of two different dinucleoside regioisomers in an approximate ratio of 2:1. Each of the regioisomers contains two nucleobase residues (two signals δ 5.89 ppm, 6.17 ppm for H5 and two signals δ 7.85 ppm, 8.09 ppm for H6 for the major regioisomer **6_ac_**, two signals δ 5.89 ppm and 6.17 ppm for H5, and two signals δ 7.82 ppm and 8.08 ppm for H6 for the minor regioisomer **6_bd_**) and two sugar residues, as shown in Figure S25. The structure of these regioisomers was confirmed by the correlating COSY, HSQC, and HMBC spectra (Figures S26–S28). The expected cross-peaks in the HMBC spectra were present in low intensity, but three binding correlations were found between C2_a_ (δ 156 ppm) and H2′_c_ (δ 4.70 ppm) for isomer **6_ac_** and three binding correlations between C2_b_ (δ 152 ppm) and H3′_d_ (δ 4.56 ppm) for isomer **6_bd_** (extended HMBC spectrum, Figure 10). Thus, the site of covalent bonding between two nucleosides in dinucleosides **6** was identified from HMBC correlations as C2-2′O for the major regioisomer **6_ac_** and C2-3′O for the minor regioisomer **6_bd_**. 

Considering that the analyzed dinucleosides **6** were the only isolated oligomeric products, as a pure mass spectral entity, we could not exclude the possibility of formation of another regioisomer of dinucleoside **6** with the C2-5′O ‘internucleoside′ linkage present in the complex mixtures of other products. It is worth noting that the observed significant shift of the resonance signal for a 3′-H proton (δ 5.54 ppm) to a lower field (to a higher frequency) indicates the possibility of formation of an intramolecular hydrogen bond involving N3_a_-3′-HO_c_ (for major C2-2′O regioisomer **6_ac_**) interactions in the seven-membered ring. Similarly, the resonance signal for a 2′-H proton (δ 5.67 ppm) in the minor C2-3′O regioisomer **6_bd_** was shifted to a lower field.

**Scheme 2:**
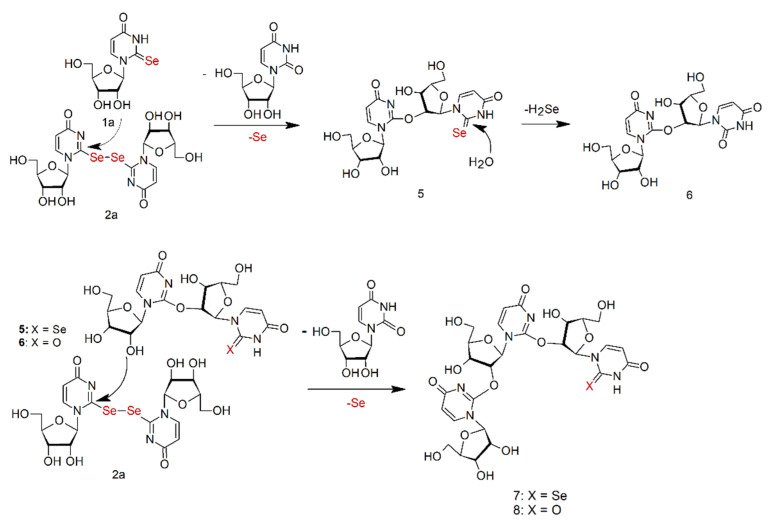
Conversion of **2** with **1** to seleno-dinucleosides **5** and seleno-trinucleosides **7** and their deselenated analogs **6** and **8**, respectively. The structures of the regioisomers with internucleoside C2-2′O bonds are shown.

In our previous studies on the oxidation of 2-selenouracil (nucleobase of **1**), we discovered that two- (Jaffe′s base) and three-ring products were formed due to the availability of the N1 nucleophilic center of Se2Ura for reaction with diselenide C2 electrophile [48]. In the present case, the nucleophilic nitrogen atom N1 of the nucleobase ring in **2** was not available, so we considered the hydroxyl groups of the ribose moiety as potential nucleophile for intermolecular condensation. Thus, the 2′-OH or 3′-OH group (possibly the first-order 5′-OH group) of **1** would intermolecularly attack the electrophilic atom C2 in diselenide **2**, thus leading to the formation of a dinucleoside with *m*/*z* 533.0418 (possibly compound **5**) and an ‘internucleoside′ bond C2-2′O or C2-3′O with the release of uridine and selenium (Scheme 2 shows an example for the 2′-OH nucleophile). Subsequent deselenation of **5** by water-assisted wash out of selenium (preferably at higher pH) should lead to the formation of the compound with *m*/*z* 469.1206 (possibly compound **6**), which was formed in the highest yield (about 8%) of all oligomeric compounds.

The trinucleoside derivatives could be the products of an intermolecular reaction, due to the nucleophilic attack of the 2′-OH or 3′-OH group of **5** or **6** (of 4-pyrimidinone riboside part) on the electrophilic carbon atom C2 of the next molecule of diselenide **2** giving rise to **7** and **8**, respectively, with the ‘internucleoside′ bond C2-2′O (or C2-3′O) (the Scheme 2 presents formation of 2′-O-regioizomers of **5**, **6**, **7** and **8**). Of course, it cannot be excluded that compound **7** was formed first and, subsequently, deselenated to **8**; although, this route may be less favorable, since only a minute amount of compound **7** was detected in the reaction mixture during the course of the reaction.

In addition, it is worth noting that lowering the pH of the reaction solution decreased the tendency to form the oligomeric side compounds, which can be explained by a lower degree of deprotonation of the compounds and, thus, lower reactivity in the nucleophilic substitution. When the reaction was carried out in water (possibly slightly acidified in the reaction course), analysis of UPLC–ESI(-)-HRMS revealed that only small amounts of selenium derivatives **5** and **7** were present. With increasing pH, the reaction proceeds much faster, which is related to the lower stability of the diselenide **2** formed. The presence of compounds with the structure of linear oligonucleotides **5**–**8**, **11**–**15**, and cyclic products **16**–**21** observed at higher pH results from the greater proportion of deprotonated forms of nucleosides (lower protonation of the nucleophilic centers) participating in the intermolecular nucleophilic substitutions.

### 2.8. Se2U Oxidation with H_2_O_2_ and Subsequent Reduction of the Resulting Products (Rescue Assay)

**Figure 11:**
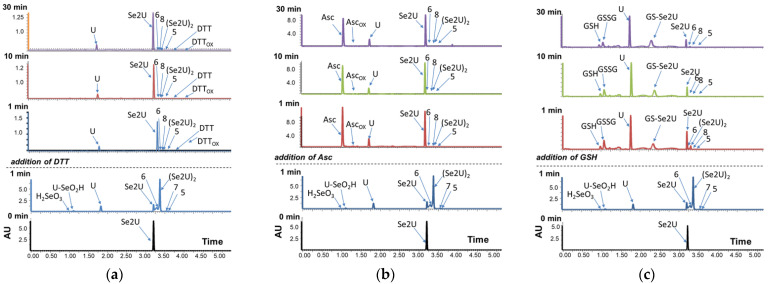
UPLC–PDA chromatographic analysis of the oxidation reaction of Se2U (**1**, 10 mM) with H_2_O_2_ (10 mM) and subsequent rescue by 10 mM reducing agent: (**a**) DTT, (**b**) ascorbic acid (Asc), (**c**) glutathione (GSH) added 1 min after the start of oxidation.

Since it is well-documented that selenium compounds are redox active cellular components, we aimed to evaluate the redox properties of selenium-modified nucleosides. Therefore, the next task was to test whether the oxidation of Se2U (**1**) is reversible, i.e., whether the intermediates of Se2U oxidation can be reduced by typical biocompatible reducing agents. To this end, a 10 mM solution of nucleoside **1** was oxidized with 10 mM H_2_O_2_ in phosphate buffer pH 7.4 for 1 min at room temperature, and then 10 mM reducing agent was added: dithiothreitol (DTT), ascorbic acid (Asc), or glutathione (GSH). The progress of the reactions was monitored for up to 30 min by UPLC–PDA–ESI(-)-HRMS, the period during which the most dynamic changes were observed. The results of these analyses are shown in Figure 11, and the UPLC–ESI(-)-MS retention times (Rt, min) and *m*/*z* values of ions corresponding to deprotonated molecules for all identified compounds are listed in Table S1.

Analysis of the reaction mixture one minute after addition of DTT showed only a trace of diselenide **2** (*m*/*z* 612.9600). The major product was Se2U (**1**) (Figure 11a), indicating that diselenide **2** was almost quantitatively reduced to substrate **1** with rapid kinetics. The presence of a small amount of uridine (**3**) was due to the oxidation of **1,** which occurred before addition to the DTT solution. Moreover, in addition to the oxidized form of dithiothreitol (DTT_OX_, *m*/*z* 150.9890), traces of dinucleoside analogs **5** (*m*/*z* 533.0418), **6** (*m*/*z* 469.1206), and trinucleoside **8** (*m*/*z* 695.1798) were detected in the reaction mixture. Thus, we demonstrated that DTT is an effective reducing agent for diselenide **2**, thus resulting in a near quantitative recovery of substrate **1**, which remained stable in the presence of DTT, until the end of reaction monitoring.

Ascorbic acid (Asc) proved to be as effective a reducing agent as DTT, and the composition of the reaction mixtures was analogous to that described above (Figure 11b). Analysis of UPLC–ESI(-)-HRMS confirmed the presence of both ascorbic acid alone (Asc, *m*/*z* 175.0240) and dehydroascorbic acid (Asc_ox_, *m*/*z* 173.0090). Traces of compounds **5**, **6**, and **8** were also identified.

An analogous reaction was also performed with glutathione (GSH), one of the most widely used natural thiol antioxidants. The intracellular concentration of GSH in the bacterium Escherichia coli is 17 mM, while in mammalian cells, it ranges from 1 to 8 mM [56,57].

The analysis performed here showed that GSH is a much weaker reducing agent than those previously tested. The intensity of the signal corresponding to uridine (**3**) was much higher, compared to the first two reactions tested (Figure 11c). Among the reaction products, as in the presence of DTT and Asc, were dinucleoside compounds **5** and **6** and a trinucleoside derivative **8**. Although the protective role of GSH against the oxidation of Se2U is only partial here, the cellular mechanism of this process is consistent with current knowledge, as it involves the formation of a mixed selenenyl sulfide (Figure S23). It is worth noting that the reduced protection of GSH, compared with DTT, can be explained by the redox potential of DTT_ox_/DTT_red_ (−327 mV), which is significantly lower than that of GSSG/GSH (−256 mV) [54]. Thanks to the analysis of UPLC-HRMS, we identified the corresponding selenenyl sulfide (GS-Se2U, *m*/*z* 612.0514) and an oxidized form of glutathione (GSSG, *m*/*z* 611.1440). It should also be emphasized that seleninic acid **4b** (U-SeO_2_H), which was present in trace amounts in the reaction mixtures before the addition of the reducing agents, was not observed in any of the solutions analyzed after the addition of the reducing agent (DTT, Asc, or GSH). It is possible that U-SeO_2_H, similar to diselenide **2**, is reduced to Se2U, as previously postulated by Hondal et al. and Prabhu et al., or it is rapidly converted to uridine and selenium (IV) oxide [32,50,58]. In any case, the amount of oxygenation product U-SeO_2_H confirms that this pathway of Se2U oxidation is not dominant and plays only a minor role, if any, in the rescue process.

## 3. Discussion

**Scheme 3:**
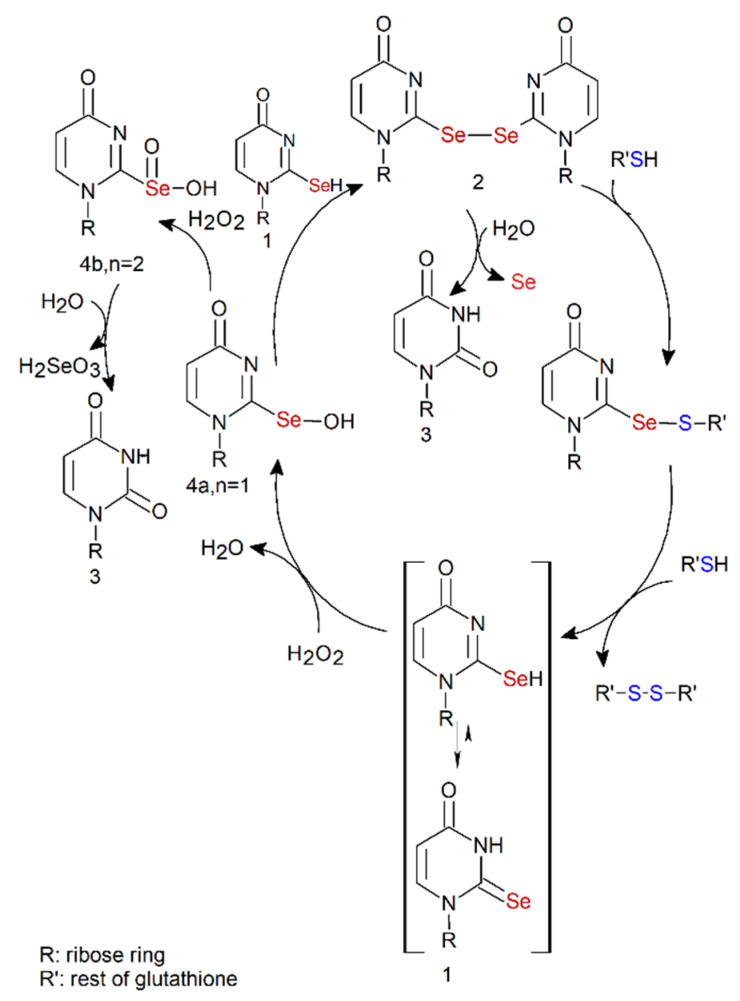
Mechanism of oxidation of Se2U in the presence of oxidative and reducing agents.

Since both S- and Se-chalcogen-containing compounds can mitigate oxidative damage in cells, we aimed to investigate the reactivity of Se2U in the presence of hydrogen peroxide and evaluate its behavior in the presence of ROS-reducing agents to prevent cellular oxidative damage. The data obtained in the presented studies showed significant differences in the course of the oxidation of Se2U and S2U with hydrogen peroxide, as Se2U (**1**) is much more susceptible to H_2_O_2_ oxidation than S2U [39]. While Se2U, upon reaction with one molar equivalent of oxidant, is completely oxidized, mainly to uridine and several minor byproducts, S2U is less susceptible to oxidation and requires an excess of hydrogen peroxide (two to three molar equivalents) to be fully utilized [36,37,38,39]. In the case of Se2U oxidation, the proposed mechanism involves the conversion of Se2U to selenenic acid **4a** (U-SeOH) in the first step (Scheme 3). Although this first oxidation product was not identified in any of our spectral and chromatographic analysis performed (probably due to its extremely high reactivity), it is assumed that this selenium derivative reacts with Se2U to form diselenide **2** [59], analogously to the process that occurs with Se2Ura [48]. The effective formation of diselenide **2** was accompanied by the presence of trace amounts of U-SeO_2_H (**4b**). This seleninic acid derivative could be considered an intermediate in the decomposition of diselenide **2** to **1** and **4a**, and a further disproportionation of **4a** to **1** and **4b**, as suggested previously [48]. On the other hand, USeO_2_H could be the product of a direct oxidation of 2-selenouridine via U-SeOH (**4a**). Moreover, the inorganic selenous acid was also detected in the oxidation reactions (Figure 2b), thus confirming the water-assisted decomposition of U-SeO_2_H (**4b**) to uridine and H_2_SeO_3_ and supporting the existence of an alternative, albeit less important, oxidation pathway of Se2U (as shown in Scheme 3). 

However, this oxidation pathway is the major oxidation process of 2-thiouridine and 2-thiouracil; it leads, via sulfenic (U-SOH), sulfinic (U-SO_2_H), and sulfonic (U-SO_3_H) intermediates, to uridine and 4-pyrimidinone ribonucleoside (H2U) [37,39,48]. 

Furthermore, in our study we have not observed the pathway described recently for the oxidation of selenoneine, which contains a selenourea motif, in which readily formed diselenide intermediate is further oxidized to the seleninic acid derivative R-SeO_2_H, and then converted to selenonic acid R-SeO_3_H, followed by deselenation to the R-H final compound [58]. In the cited selenoneine work and as it was earlier suggested by the Hondal group for 5-carboxy-2-selenouracil (c5Se2Ura) [32], seleninic acid derivatives R-SeO_2_H can be reversibly reduced to the keto selenium substrates, a phenomenon we did not observe here, and the mechanism of this suggested reversible process has not been clearly shown to date [32,58].

Importantly, we showed that diselenide **2** could be effectively transformed to parent **1** under reducing conditions, as tested with DTT, ASC, and GSH (Scheme 3). We report here, for the first time, a direct formation of a mixed selenenyl sulfide between glutathione (GSH) and 2-selenouridine that could undergo facile reaction with the second molecule of GSH, thus resulting in recovered 2-selenouridine and oxidized form of glutathione (GS-SG). 

In contrast, 2-thiouridine does not oxidize to its disulfide derivative (S2U)_2_ [37,39], and the sulfur oxoacids intermediate oxidation products of S2U under DTT, ASC, and GSH treatment are irreversible moieties. It should be mentioned that the formation of diselenide, as well as oligomeric minor products, obviously does not occur at the level of tRNA when Se2U is the component of the oligonucleotide chain. However, one cannot exclude the possibility that Se2U tRNA is metabolized to nucleosides by nucleolytic degradation; then, 2-selenouridine is converted to the described products under oxidative stress conditions. 

To our knowledge, there are no published reports regarding the formation of oligomeric compounds resulting from the properties of 2-selenouridine and uridine diselenide. The conversion of **2** with **1** to seleno-dinucleosides **5** and seleno-trinucleosides **7**, as well as their deselenated analogs **6** and **8** (see Scheme 2), which lead to new oligomeric nucleosides, are only weakly related to the function of tRNA in the cell (at least in our opinion), but they are of interest because of the unique structural oligonucleoside scaffold formation.

The different redox properties of organo-selenium and organo-thio compounds are the result of the different physicochemical properties of sulfur and selenium elements [59]. Selenium is a more polarizable element than sulfur and forms weaker covalent bonds, making selenium compounds more reactive under both nucleophilic and electrophilic substitution conditions. Thus, unlike sulfur analogs, the higher oxidation forms of selenium compounds are relatively unstable. As a heavier element that forms longer bonds, the selenium atom also has a lower ability to form all types of π-bonds. The consequence of selenium′s less attractive valence electrons is its stronger nucleophilic character, which allows selenium compounds to react more rapidly with reactive oxygen species than sulfur analogs; at the same time, the dipole character of the Se^+^-O^-^ bond enables their much faster reduction, compared to S-oxides. These remarkable properties of organo-selenium compounds allow us to maintain a kind of equilibrium between oxidation and reduction states. The replacement of sulfur atoms with selenium atoms in natural molecules, such as enzymes, often protects biomolecules from permanent damage due to oxidative stress. 

**Figure 12:**
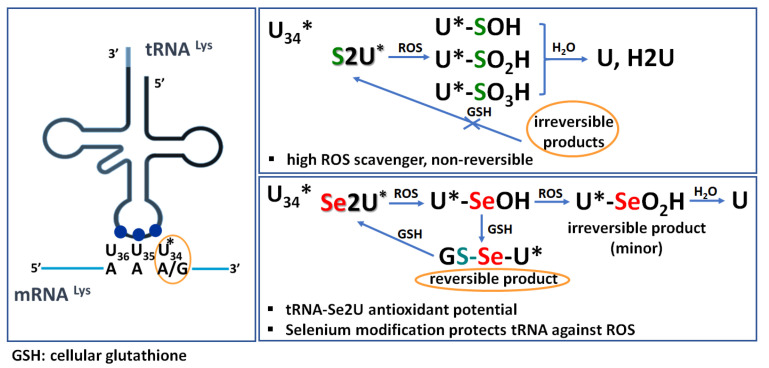
Schematic difference between sulfur- and selenium-modified uridines (R5S2U and R5Se2U) found in wobble position of transfer RNAs in the presence of redox-reactive agents (reactive oxygen species ROS and reducing agents as glutathione).

It is also possible that the 2-selenouridine component of tRNA has antioxidant potential (Figure 12) in cells. Our results indicate that 2-selenouridine is converted to diselenide (Se2U)_2_ via the selenenic acid U-SeOH reactive precursor. We hypothesize that, on the tRNA level, 2-selenouridine might be oxidized by ROS to selenenic acid tRNA-U*-SeOH, which can interact predominantly with cellular selenides or sulfides, e.g., with cellular glutathione (GSH), thus forming mixed selenenyl sulfide as an intermediate GS-Se2U*-tRNA, which, in turn, can be reduced with GSH to the native tRNA-Se2U*. This reversible oxidation/reduction mechanism of the 2-selenouridine component of tRNA is suggested to contribute to the antioxidant properties of selenium-modified tRNAs during oxidative stress in bacterial cells. 

We assume that the formation of the corresponding tRNA diselenides (analogously to uridine diselenide) at the tRNA level is not credible because of the very low tRNA-Se2U* concentration and, probably, steric limitations. When formed, selenic acid U*-SeOH can be further oxidized to seleninic acid U*-SeO_2_H at higher ROS concentrations. This intermediate, which resembles the sulfinic acid form of 2-thiouridine, is susceptible to water-assisted decomposition to uridine and has no potential to contribute to the selenium redox-active properties of the transfer RNA.

## 4. Materials and Methods

### 4.1. NMR Spectroscopy 

^1^H, ^13^C, COSY, HMQC, and HMBC NMR spectra were recorded on a Bruker Avance 700 MHz spectrometer. The NMR spectra for ^1^H and ^13^C were recorded at 700 and 176 MHz, respectively. Chemical shifts (δ) were reported in ppm, relative to TSP (an internal standard), for ^1^H and ^13^C. The signal multiplicities are described as s (singlet), d (doublet), t (triplet), q (quartet), and m (multiplet). Coupling constants were reported in hertz (Hz). ^77^Se NMR measurements were performed using a Bruker Avance III 500 spectrometer (Bruker, Zurich, Switzerland), operating at 95 MHz. Chemical shifts (δ) were reported in ppm, relative to H_2_SeO_3_ (an internal standard).

### 4.2. Ultra-Performance Liquid Chromatography Coupled with a High-Resolution Mass Spectrometry and Photodiode Array Detection (UPLC–PDA–ESI(–)-HRMS)

The identification of the reaction products was carried out by ACQUITY UPLC I-Class chromatography system equipped with a photodiode array detector with a binary solvent manager (Waters Corporation, Milford, MA, USA), coupled with SYNAPT G2-Si mass spectrometer, equipped with an electrospray source and quadrupole time-of-flight mass analyzer (Waters Corp., Milford, MA, USA). For the chromatographic separation of analyte an Acquity HSS T3 1.8 μm column (100 × 2.1 mm) (Waters Corporation, Milford, MA, USA), thermostated at 30 °C was used. Mobile phases consisted of 10 mM CH_3_COONH_4_ (solvent A) and 50% CH_3_CN in 10 mM CH_3_COONH_4_ (solvent B). A gradient program was employed as follows: 10% B (0–1.0 min), 10–95% B (1.0–3.5 min), 95–99% B (3.5–4.0 min), 95–10% B (4.0–4.1 min), and 10–10% B (4.1–6 min). The flow rate was 0.2 mL/min, and the injection volume was 1 μL. For mass spectrometric detection the electrospray source was operated in a negative, high-resolution mode at a 50,000 FWHM resolving power for the TOF analyzer. The optimized source parameters were: capillary voltage 3 kV, cone voltage 20 V, source temperature 90 °C, desolvation gas (nitrogen) flow rate 600 L/h with the temperature 350 °C, and nebulizer gas pressure 6.5 bar. To ensure accurate mass measurements, data were collected in centroid mode and mass was corrected during acquisition using leucine enkephalin solution as an external reference (Lock-SprayTM), which generated reference ion at *m*/*z* 554.2615 ([M-H]^−^) in negative ESI mode. Mass spectra was recorded over an *m*/*z* range of 100 to 1200. For collision-induced fragmentation experiments, argon was used as collision gas, and the collision energy was ramped from 15 to 35 eV. The PDA spectra were measured over the wavelength range of 210–400 nm in steps of 1.2 nm. The results of the measurements were processed using the MassLynx 4.1 software (Waters) incorporated with the instrument. 

### 4.3. Chemistry

#### 4.3.1. Materials and Reactions

Anhydrous solvents, such as dichloromethane (DCM), methanol (MeOH), ethanol (EtOH), and acetonitrile (MeCN), were prepared according to standard procedures. Glutathione and DL-dithiothreitol were purchased from Serva and ascorbic acid was purchased from Aldrich Company. Selenium-77 (99.6% atom Se-77) was purchased from Icon Isotopes.

#### 4.3.2. Synthesis of 2-selenouridine

The synthesis of 2-selenouridine (of ^77^Se or naturally occurring selenium isotopes) was carried out according to the published procedures with slight improvements [26,60]. The detailed procedure is described in the Supplementary Materials. 

#### 4.3.3. H/^77^Se NMR Analysis of the Oxidation Experiments of 1 

A solution of **1** was prepared either in 67 mM phosphate buffer (pH 7.4, pH 8.0, and pH 5.0) or deionized water (using D_2_O). The first ^1^H NMR spectrum was recorded to determine the initial point. Compound **1** was treated with 0.5, 1, or 10 equivalents of H_2_O_2_. The reactions were monitored by NMR, and spectra were recorded after 1 or 2 min, 5 min, 20 min, 30 min, 60 min, 120 min, and 24 h. The reaction conditions and yields were determined. The reaction conditions and yields of the products are given in Table 1.

#### 4.3.4. LC–MS Analysis of the Oxidation Assays of 1

A 10 mM solution of **1** was prepared in 67 mM phosphate buffer (pH 7.4, pH 8.0, pH 5.0) or MilliQ water, and then treated with 0.5, 1, or 10 equivalents of H_2_O_2_. The reactions were monitored by LC–MS and spectra were acquired after 1min, 10 min, 30 min, 60 min, 120 min, and 24 h.

#### 4.3.5. Rescue Assay Conditions of 1 for LCMS Analysis

A 10 mM solution of **1** was prepared in 67 mM phosphate buffer at pH 7.4. A total of one equivalent of H_2_O_2_ was added to the solution, and then the reaction was allowed to proceed for 1 min at room temperature. After 1 min, one equivalent of either DTT (dithiothreitol), Asc (ascorbic acid), or GSH (glutathione) was added to the solutions, and the reactions were monitored by LC–MS. Spectra were acquired after 1min, 10 min, 30 min, 60 min, 120 min, and 24 h.

#### 4.3.6. General Approach for Analysis of the Course of Oxidation of 2-selenouridine (Se2U, 1) and Identification of the Reaction Products

To obtain data on the course of oxidation of 2-selenouridine (Se2U, **1**), the nucleoside substrate was reacted in 10, 1, or 40 mM solutions in phosphate buffer at pH 5.0, 7.4, or 8.0, water at room temperature (rt.), or at 10 °C with hydrogen peroxide (H_2_O_2_) in molar ratios of 1:0.5, 1:1, or 1:10. The detailed list of experiments and content of each reaction product can be found in Table 1. The reaction course (from 1 min to 24 h) and structural data of the intermediates were determined by ^1^H NMR, ^77^Se NMR, RP-HPLC, and UPLC–PDA–ESI(-)-HRMS analyses. The relative content of the identified products was calculated from integrations of the ^1^H NMR signals for the non-exchangeable H5, H6, and H1′ protons (δ-range 8.8–5.2 ppm) in the presence of the internal standard (5 mM thymine). The UPLC–ESI(-)-MS retention times (Rt, min), *m*/*z* values of ions corresponding to deprotonated molecules, λ_max_ in UV/VIS spectra (nm), and ^1^H NMR chemical shifts (δ, ppm) for H5, H6, and H1′ protons and coupling constants *J_H6-H5_* and *J_H1′-H2′_* for all identified compounds are listed in Table 2. Spectral data for all identified compounds can be found in the Supplementary Materials (Figures S1–S28).

## Figures and Tables

**Table 1:** Detailed listing of oxidation experiments for Se2U (#1-13), with the content of each reaction product (%). Reactions of Se2U (10 mM or otherwise defined in the footnote) with hydrogen peroxide in molar ratios of 1:1, 1:0.5, and 1:10 were carried out either in phosphate buffer pH 5.0, 7.4, and 8.0, water at room temperature (rt.), or at 10 °C. The content of the reaction products was determined by integration of the ^1^H NMR signals for the non-exchangeable H5, H6, and H1′ protons (δ-range 8.8–5.2 ppm) in the presence of the internal standard (5 mM thymidine).

No. (#)	Se2U	Molar Ratio Se2U:H_2_O_2_	pH	T [°C]	Product Content [%]							
					1	2	3	4	5	6	7	8
1	Se2U **1**	1:1	7.4	rt.	-	*+*	71	+ (*n* = 2)	+	8	+	+
2		1:1	8.0	rt.	-	*+*	53	*-*	+	5	+	+
3		1:1	5.0	rt.	-	*+*	100	+ (*n* = 1, 2)	-	-	-	-
4		1:1	water	rt.	<1	*+*	97	+ (*n* = 1, 2)	+	1	+	+
5		1:10	7.4	rt.	-	*+*	100	+ (*n* = 2)	+	-	+	-
6		1:10	water	rt.	-	*+*	100	+ (*n* = 1, 2)	+	<1	+	-
7		1:10	water	10	-	*+*	99	+ (*n* = 1, 2)	-	<1	-	-
8		1:0.5	7.4	rt.	28	*+*	27	*-*	+	4	+	+
9		1:0.5	7.4	10	19	*-*	22	*-*	-	6	-	-
10		1:1	7.4	10	-	*-*	59	*-*	-	8	-	-
11		1:0.5	water	10	11	*+*	87	*-*	+	<1	+	+
12	^77^Se2U	1^1^:0.5	water	10	+	+		+				
13		1^1^:0.5	7.4	10	+							

^1^ For ^77^Se NMR the starting ^77^Se2U was used in 40 mM and H_2_O_2_ in 20 mM concentration; # index for the experiment number.

**Table 2:** UPLC–PDA–ESI(-)-HRMS and ^1^H NMR data of **1** and organic products **2**–**8** of the oxidation reaction of Se2U with H_2_O_2_. For all identified compounds, the UPLC retention time (Rt, min) and *m*/*z* data (atomic mass unit to its formal charge ratio) for [M-H]^−^ in negative mode, the maximum wavelength λ_max_ in UV/VIS spectra (nm) and ^1^H NMR chemical shifts (ppm) of H5 and H6 protons and their coupling constant *J_H_*_6*H*5_ is given.

Compound		UPLC–PDA–ESI(–)-HRMS				UV	^1^H NMR				
		Elemental Composition	Rt [min]	*m*/*z* [M-H]^−^		λ_max_	H6 [ppm]	H5 [ppm]	H1′ [ppm]	*J_H_*_6*-H*5_	*J_H_*_1*′-H*2*′*_
				Calcd	Found						
**1**	Se2U	C_9_H_12_N_2_O_5_Se	3.26	306.9833	306.9834	307	8.14	6.28	6.83	8.2	2.6
**2**	(USe2U)_2_	C_18_H_22_N_4_O_10_Se_2_	3.43	612.9592	612.9600	241	8.16	6.34	6.11	7.6	5.3
**3**	U	C_9_H_12_N_2_O_6_	1.84	243.0617	243.0623	261	7.78	5.82	5.80	8.1	4.7
**4a**	U-SeOH	C_9_H_12_N_2_O_6_Se		322.9782	322.9781	-	8.18	6.31	6.19	7.6	3.1
**4b**	U-SeO_2_H	C_9_H_12_N_2_O_7_Se	1.14	338.9731	338.9720	249	8.13	6.26	5.86	7.7	4.4
**5**		C_18_H_22_N_4_O_10_Se	3.60	533.0423	533.0418	238/306					
**6**		C_18_H_22_N_4_O_11_	3.37	469.1207	469.1206	235/252	8.16	6.25	6.07	7.6	3.5
							7.92	5.97	6.10	8.1	5.8
**7**		C_27_H_32_N_6_O_15_Se	3.42	759.1020	759.1020	243/307	-	-	-	-	-
**8**		C_27_H_32_N_6_O_16_	3.42	695.1796	695.1798	234/250	-	-	-	-	-

^1^H NMR data not available.

**Table 3:** The content of uridine **3** in the reaction mixture (in %).

Conditions/Reaction Number	Content of 3 after 2 min [%]
pH 5/3	5
Water/4	7
pH 7.4/1	35
pH 8/2	45

**Table 4:** UPLC–HRMS identification products of cyclization/oligomerization.

Compound	n	X	UPLC–PDA–ESI(-)-HRMS		
			Elemental Composition	[M-H] *m*/*z*	
				Calcd	Found
**Products of oligomerization**					
**5**	0	Se	C_18_H_21_N_4_O_10_Se	533.0423	533.0424
**6**	0	O	C_18_H_21_N_4_O_11_	469.1207	469.1212
**7**	1	Se	C_27_H_31_N_6_O_15_Se	759.1013	759.1013
**8**	1	O	C_27_H_31_N_6_O_16_	695.1797	695.1800
**11**	2	Se	C_36_H_41_N_8_O_20_Se	985.1602	985.1606
**12**	2	O	C_36_H_41_N_8_O_21_	921.2386	921.2386
**13**	3	O	C_45_H_51_N_10_O_26_	1147.2976 573.1449 ^a^	1147.3007 573.1464 ^a^
**14**	4	O	C_54_H_61_N_12_O_31_	1373.3566 686.1744 ^a^	1373.3599 686.1730 ^a^
**15**	5	O	C_63_H_72_N_14_O_36_	799.2039 ^a^	799.2028 ^a^
**Products of cyclization**					
**16**	0	-	C_18_H_19_N_4_O_10_	451.1101	451.1103
**17**	1	-	C_27_H_29_N_6_O_15_	677.1691	677.1686
**18**	2	-	C_36_H_39_N_8_O_20_	903.2281	903.2280
**19**	3	-	C_45_H_49_N_10_O_25_	1129.2870	1129.2883
**20**	4	-	C_54_H_59_N_12_O_30_	1355.3460	1355.3461
**21**	5	-	C_63_H_69_N_14_O_35_	790.1985 ^a^	790.1981 ^a^

^a^ Peak corresponds to the double charged ions.

## Supplementary Materials

The following supporting information can be downloaded at: https://www.mdpi.com/article/10.3390/ijms23147973/s1.
