# Supplementary material for: 2-Selenouridine, a Modified Nucleoside of Bacterial tRNAs, Its Reactivity in the Presence of Oxidizing and Reducing Reagents
Source: Int J Mol Sci. 2022 Jul 19;23(14):7973. doi: 10.3390/ijms23147973 (PMC9325004; doi:10.3390/ijms23147973)
Supplement: Supplementary file 1 [file ijms-23-07973-s001.zip › ijms-1815039-supplementary.pdf]

SUPPORTING INFORMATION

2-Selenouridine, a Modified Nucleoside of Bacterial tRNAs, Its Reactivity in the Presence of Oxidizing and Reducing Reagents

Katarzyna Kulik [1,*], Klaudia Sadowska [2], Ewelina Wielgus [1], Barbara Pacholczyk-Sienicka [2], Elzbieta Sochacka [2] and Barbara Nawrot [1]

[1] Centre of Molecular and Macromolecular Studies, Polish Academy of Sciences, Sienkiewicza 112, 90-363 Lodz, Poland; ms@cbmm.lodz.pl (E.W.); bnawrot@cbmm.lodz.pl (B.N.)
[2] Institute of Organic Chemistry, Lodz University of Technology, Zeromskiego 116, 90-924 Lodz, Poland; klaudia.sadowska@dokt.p.lodz.pl (K.S.); barbara.pacholczyk@p.lodz.pl (B.P.-S.); elzbieta.sochacka@p.lodz.pl (E.S.)
* Correspondence: kpieta@cbmm.lodz.pl; Tel.: +48-(42)-68-03-215

Table of contents:

1. **Chemistry**
a. Synthesis of 2-selenouridine (Se2U, **1**)
b. Synthesis of 2-selenouridine (Se2U, **1**) labelled ^{77}Se isotope selenium

2. **Spectral and mass spectrometry analysis of products**

Figure S1. ESI(−)-HRMS analysis and UV spectrum of **1**
Figure S2. ^{1}H NMR (200 MHz, D_2O) of **1**
Figure S3. ESI(-)-HRMS analysis of **1** labelled ^{77}Se isotope selenium
Figure S4. ^{1}H NMR (500 MHz, D_2O) spectrum of **1** labelled ^{77}Se isotope selenium
Figure S5. ESI(−)-HRMS analysis and UV spectrum of **2**
Figure S6. ESI(−)-HRMS analysis of **4a** , n=1
Figure S7. ESI(−)-HRMS analysis and UV spectrum of **4b** , n=2
Figure S8. ESI(−)-HRMS analysis and UV spectrum of **5**
Figure S9. ESI(−)-HRMS analysis of and UV spectrum of **6**
Figure S10. ESI(−)-HRMS analysis and UV spectrum of **7**
Figure S11. ESI(−)-HRMS analysis of and UV spectrum of **8**
Figure S12. ESI(−)-HRMS analysis of **11**
Figure S13. ESI(−)-HRMS analysis of **12**
Figure S14. (A)ESI(−)-HRMS analysis of **13**; (B) ESI(-) MS of **13** with peaks correspond the double charged ions
Figure S15. (A)ESI(−)-HRMS analysis of **14**; (B) ESI(-) MS of **14** with peaks correspond the double charged ions
Figure S16. ESI(−)-MS of **15** with peaks correspond the double charged ions
Figure S17. ESI(−)-HRMS analysis of **16**
Figure S18. ESI(−)-HRMS analysis of **17**
Figure S19. ESI(−)-HRMS analysis of **18**
Figure S20. ESI(−)-HRMS analysis of **19**
Figure S21. ESI(−)-HRMS analysis of **20**

Figure S22. ESI(−)-MS of **21** with peaks correspond the double charged ions
Figure S23. ESI(−)-HRMS analysis and UV spectrum of **22**
Figure S24. EI (electron impact) mass spectrum of product released as red precipitate
Figure S25. ^{1}H NMR (700 MHz, D_2O) spectrum of dinucleoside **6**
Figure S26. ^{1}H-^{1}H COSY (700 MHz, D_2O) of **6**
Figure S27. ^{1}H-^{13}C HSQC (700 MHz, D_2O) of **6**
Figure S28. ^{1}H-^{13}C HMBC (700 MHz, D_2O) of **6**
Table S1. UPLC-PDA-ESI (-)-HRMS products identified during rescue assay

3. **Time course of the oxidation of Se2U (1) by hydrogen peroxide monitored by NMR spectroscopy and LCMS**

Figure S29. ^{1}H NMR analysis of the reaction mixtures for oxidation of Se2U (**1**, 10 mM) with H_2O_2 (100 mM) in 67 mM phosphate buffer pH 7.4, at room temperature.
Figure S30. UPLC-PDA chromatographic analysis of the reaction mixtures for oxidation of Se2U (**1**, 10 mM) with H_2O_2 (100 mM) in 67 mM phosphate buffer pH 7.4 , at room temperature.
Figure S31. ^{1}H NMR analysis of the reaction mixtures for oxidation of Se2U (**1**, 10 mM) with H_2O_2 (10 mM) in 67 mM phosphate buffer pH 8.0, at room temperature.
Figure S32. ^{1}H NMR analysis of the reaction mixtures for oxidation of Se2U (**1**, 10 mM) with H_2O_2 (10 mM) in 67 mM phosphate buffer pH 5.0, at room temperature.
FigureS33. ^{1}H NMR analysis of the reaction mixtures for oxidation of Se2U (**1**, 10 mM) with H_2O_2 (10 mM) in water, at room temperature
Figure S34. ^{1}H NMR analysis of the reaction mixtures for oxidation of Se2U (**1**, 10 mM) with H_2O_2 (100 mM) in water, at room temperature
Figure S35. UPLC-PDA chromatographic analysis of the reaction mixtures for oxidation of Se2U (**1**, 10 mM) with H_2O_2 (10 mM) in deionized water, at room temperature
Figure S36. UPLC-PDA chromatographic analysis of the reaction mixtures for oxidation of Se2U (**1**, 10 mM) with H_2O_2 (100 mM) in deionized water, at room temperature
Figure S37. ^{1}H NMR analysis of the reaction mixtures for oxidation of Se2U (**1**, 10 mM) with H_2O_2 (5 mM) in 67 mM phosphate buffer pH 7.4, at 10 °C
Figure S38. ^{1}H NMR analysis of the reaction mixtures for oxidation of Se2U (**1**, 10 mM) with H_2O_2 (10 mM) in 67 mM phosphate buffer pH 7.4, at 10 °C
Figure S39 ^{1}H NMR analysis of the reaction mixtures for the oxidation of Se2U (**1**, 40 mM) with H_2O_2 (20 mM) in phosphate buffer pH 7.4 at 10 °C.

1a. Synthesis of 2-selenouridine (Se2U, **1**)

2-Selenouridine (**1**) was obtained according to the previously described procedure [1]. ^{1}H NMR (500 MHz, D_2O) δ 8.04 (dd, J = 8.2, 1H), 6.58 (s, 1H), 6.16 (d, J = 8.0 Hz, 1H), 4.30 (s, 1H), 4.07 (s, 2H), 3.88 (dd, J = 12.4, 1H), 3.73 (dd, J = 12.4, 1H). HRMS (ESI); calcd. for $C_9H_{11}N_2O_5Se$ $[M-H]^-$ 306.9833, found 306.9834. Spectral data are given Figures S1 and S2.

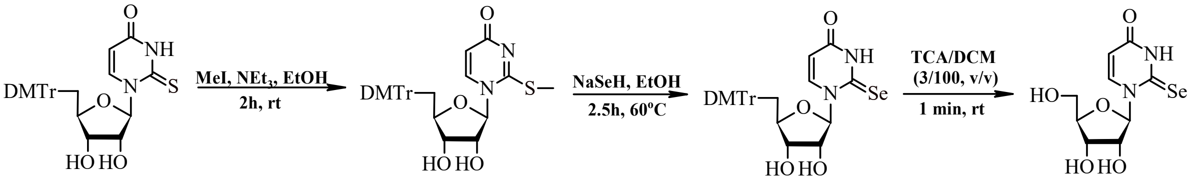

1b. Synthesis of 2-selenouridine (Se2U, **1**) labelled ^{77}Se isotope selenium

2-Selenouridine (**1**) was obtained as mentioned above with using the NMR-active ^{77}Se isotope of selenium to generate the sodium hydroselenide (NaHSe). ^{1}H NMR (500 MHz, D_2O) δ 8.01 (d, J = 8.4, 1H), 6.55 (m, 1H), 6.13 (d, J = 8.0 Hz, 1H), 4.22 (q, J = 2.6 Hz, 1H), 4.04 (d, J=2.5Hz, 2H), 3.85 (dd, J = 13.1, 1.6 Hz, 1H), 3.70 (dd, J = 13.1, 2.5 Hz, 1H). ^{77}Se NMR (95 MHz, D_2O) δ 353.79. HRMS (ESI); calcd. for $C_9H_{11}N_2O_5{}^{77}Se$ $[M-H]^-$ 303.9867, found 303.9866. Spectral data are given Figures S3 and S4.

2. Spectral and mass spectrometry analysis of products

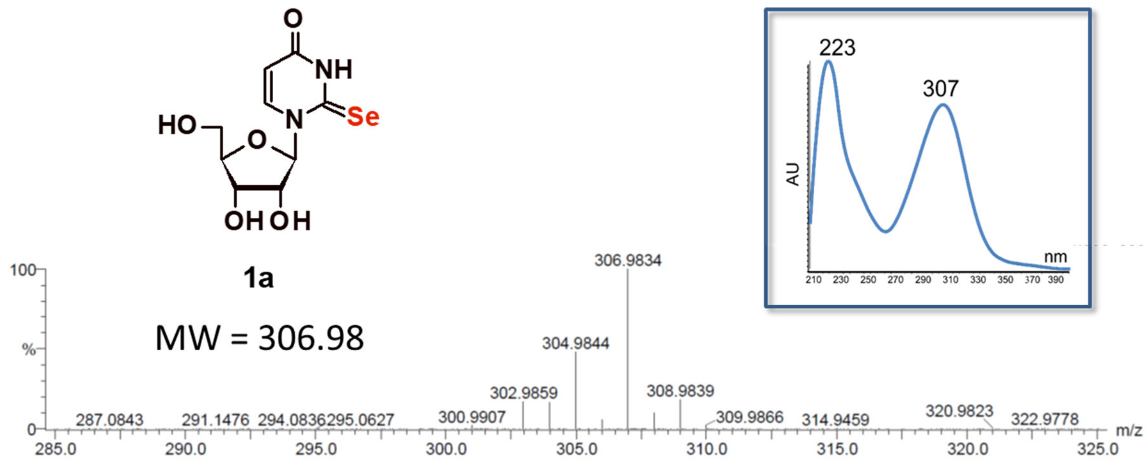

Minimum:				-1.5				
Maximum:		5.0	5.0	90.0				
Mass	Calc. Mass	mDa	PPM	DBE	i-FIT	Norm	Conf(%)	Formula
306.9834	306.9833	0.1	0.3	6.5	630.2	n/a	n/a	C9 H11 N2 O5 Se

Figure S1. ESI(−)-HRMS analysis and UV spectrum of **1.**

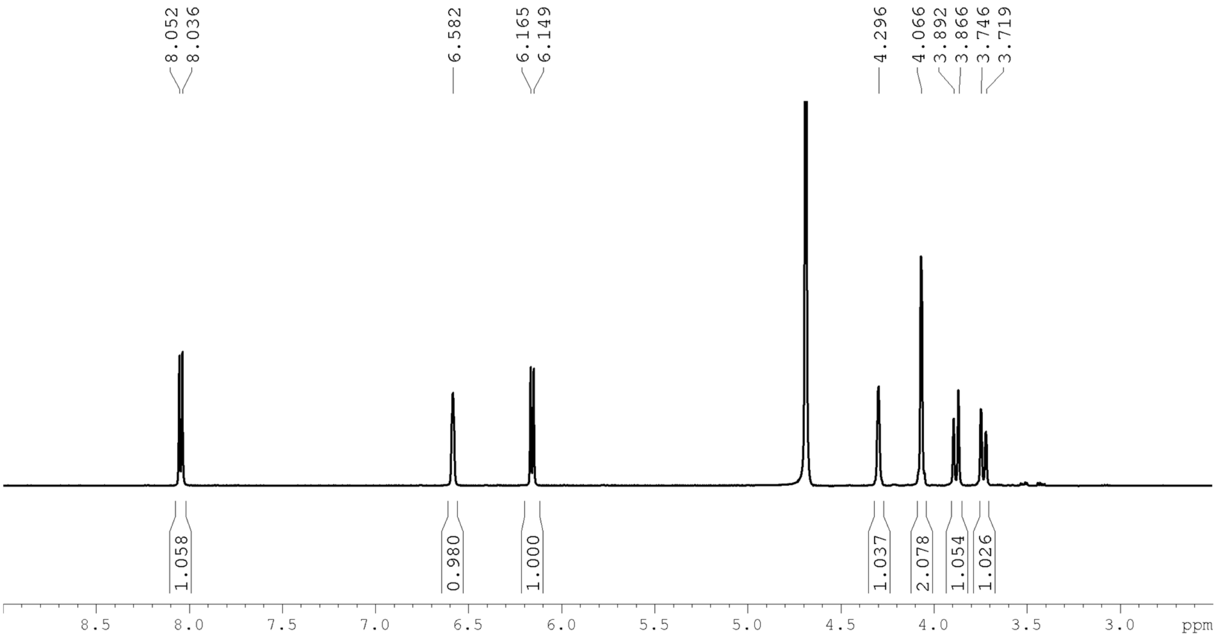

Figure S2. ^{1}H NMR (500 MHz, D_2O) of **1.**

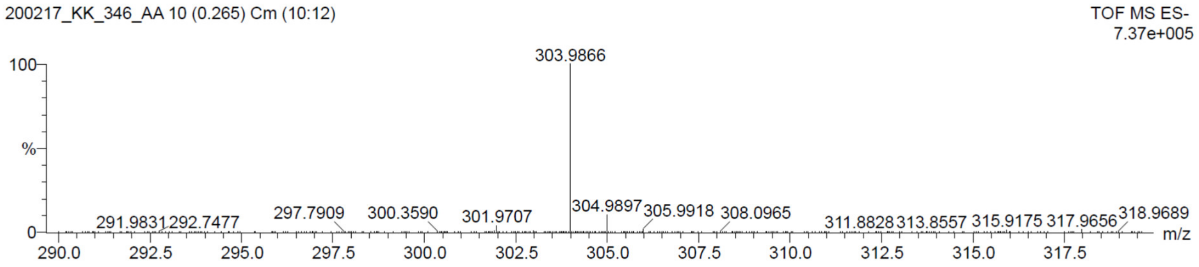

Minimum:				-1.5				
Maximum:		5.0	5.0	90.0				
Mass	Calc. Mass	mDa	PPM	DBE	i-FIT	Norm	Conf(%)	Formula
303.9866	303.9867	-0.1	-0.3	6.5	1055.3	0.000	99.99	C9 H11 N2 O5 77Se

Figure S3. ESI(-)-HRMS analysis of **1** labelled ^{77}Se isotope selenium.

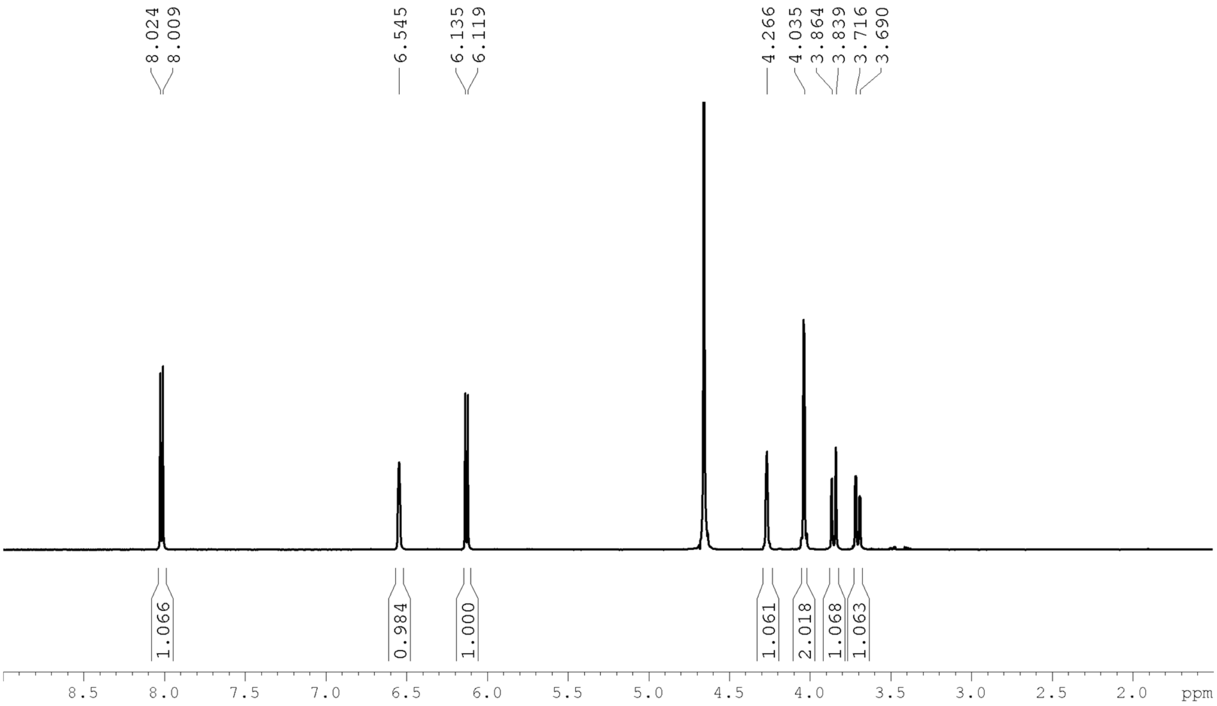

Figure S4. ^{1}H NMR (500 MHz, D_2O) spectrum of **1** labelled ^{77}Se isotope selenium.

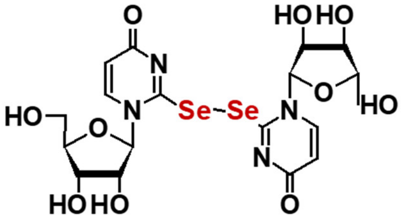

2a

MW = 612.96

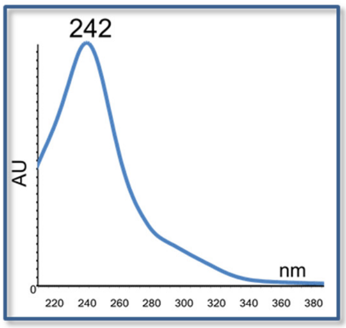

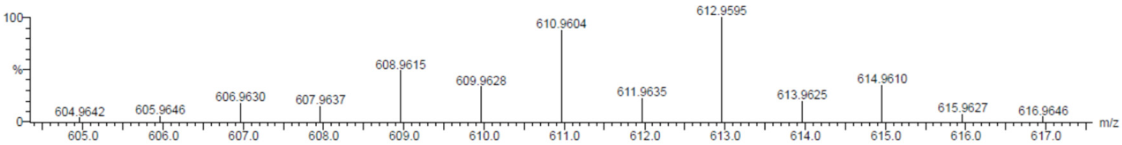

Minimum:				-10.0				
Maximum:		15.0	5.0	60.0				
Mass	Calc. Mass	mDa	PPM	DBE	i-FIT	Norm	Conf(%)	Formula
612.9595	612.9588	0.7	1.1	12.5	133.6	0.691	50.12	C18 H21 N4 O10 Se2

Figure S5. ESI(−)-HRMS analysis and UV spectrum of **2.**

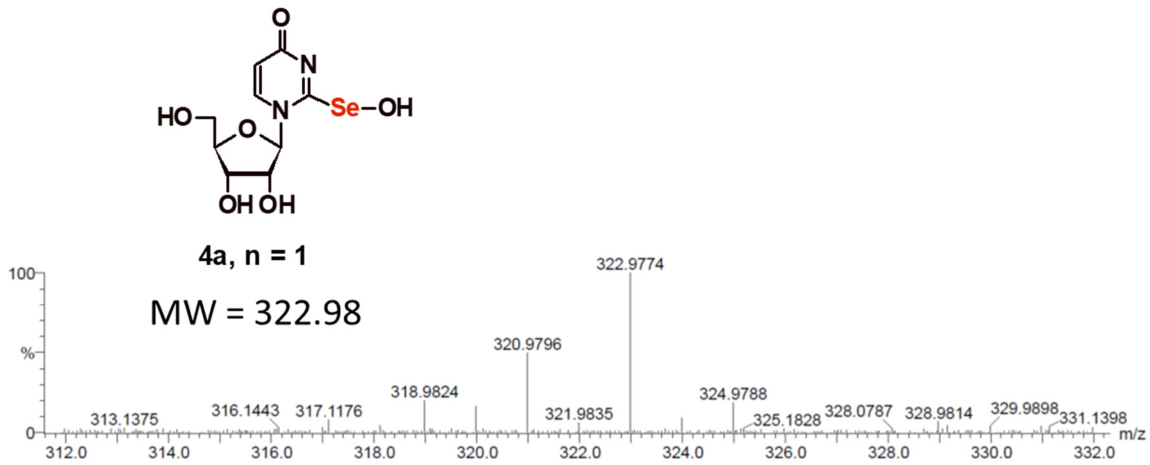

Minimum:				-1.5					
Maximum:		5.0	5.0	90.0					
Mass	Calc. Mass	mDa	PPM	DBE	i-FIT	Norm	Conf(%)	Formula	
322.9774	322.9782	-0.8	-2.5	6.5	1266.3	n/a	n/a	C9 H11 N2 O6 Se	

Figure S6. ESI(−)-HRMS analysis of **4a**, n=1.

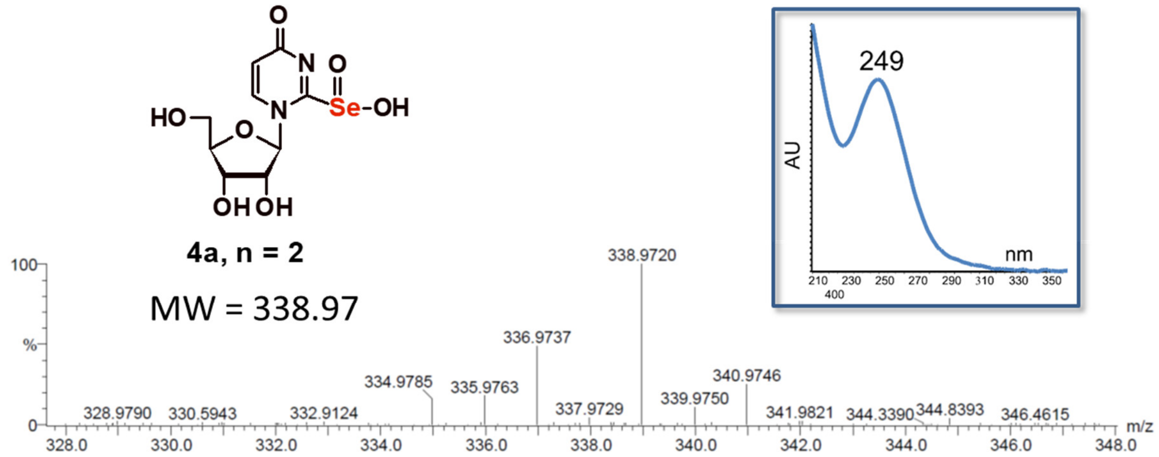

Minimum:				-1.5				
Maximum:		5.0	5.0	90.0				
Mass	Calc. Mass	mDa	PPM	DBE	i-FIT	Norm	Conf(%)	Formula
338.9720	338.9731	-1.1	-3.2	6.5	292.9	n/a	n/a	C9 H11 N2 O7 Se

Figure S7. ESI(−)-HRMS analysis and UV spectrum of **4b ,** n=2.

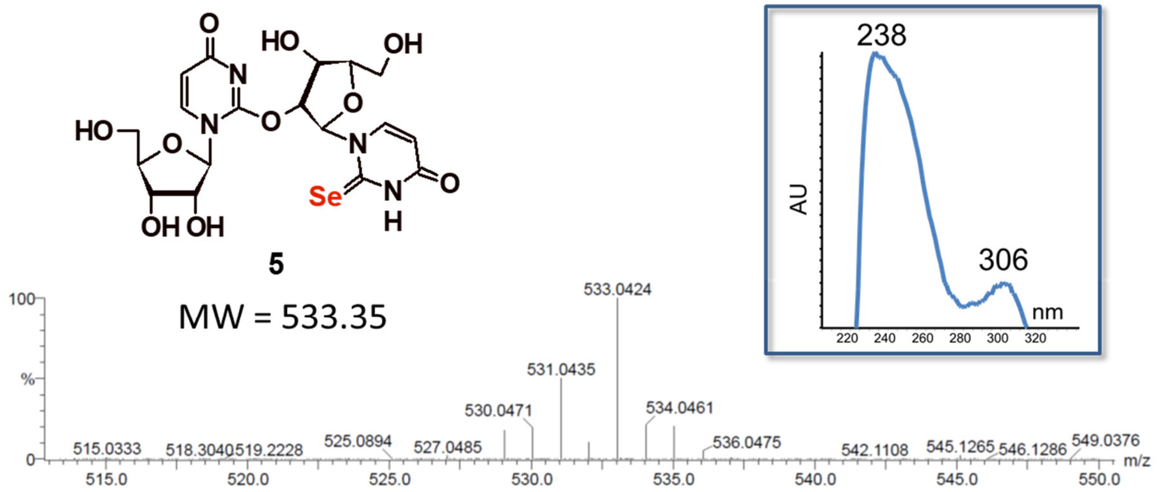

Minimum:				-1.5				
Maximum:		5.0	5.0	80.0				
Mass	Calc. Mass	mDa	PPM	DBE	i-FIT	Norm	Conf(%)	Formula
533.0424	533.0423	0.1	0.2	11.5	912.8	n/a	n/a	C18 H21 N4 O10 Se

Figure S8. ESI(−)-HRMS analysis and UV spectrum of **5.**

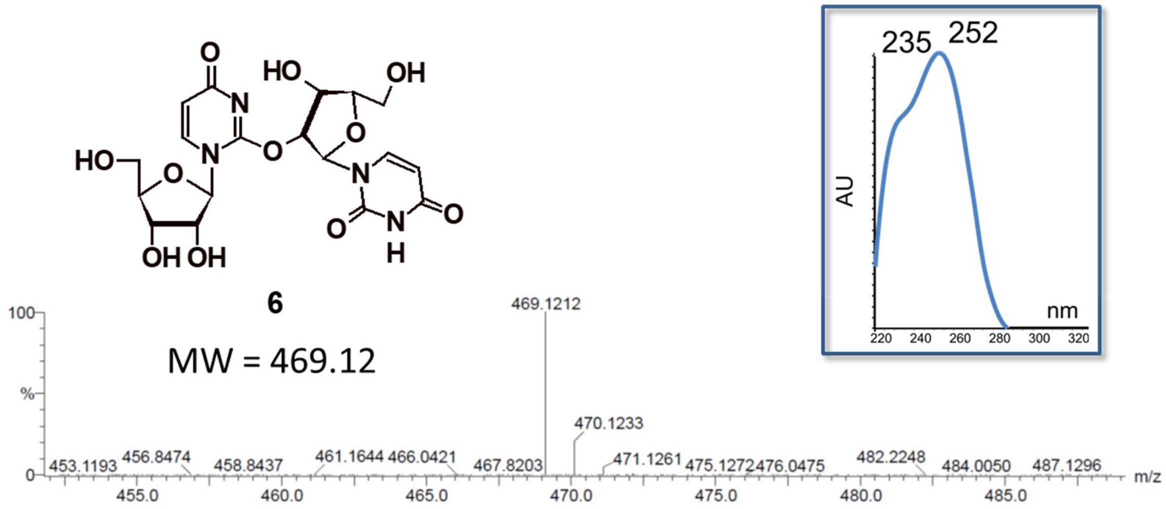

Minimum:				-1.5				
Maximum:		5.0	5.0	80.0				
Mass	Calc. Mass	mDa	PPM	DBE	i-FIT	Norm	Conf(%)	Formula
469.1212	469.1207	0.5	1.1	10.5	721.9	n/a	n/a	C18 H21 N4 O11

Figure S9. ESI(−)-HRMS analysis of and UV spectrum of **6.**

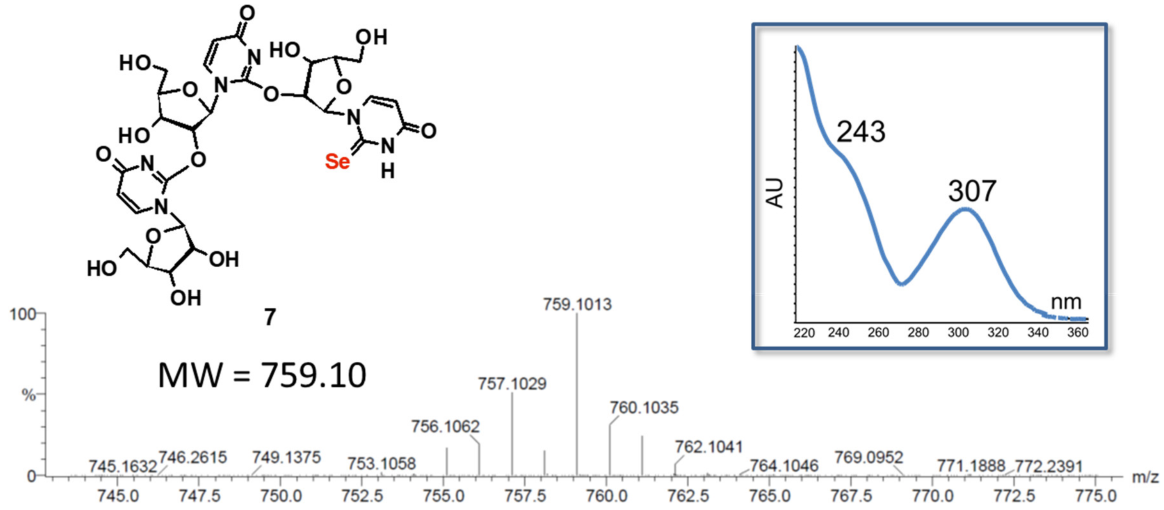

Minimum:				-1.5				
Maximum:		5.0	5.0	80.0				
Mass	Calc. Mass	mDa	PPM	DBE	i-FIT	Norm	Conf(%)	Formula
759.1013	759.1013	0.0	0.0	16.5	879.8	0.000	99.99	C27 H31 N6 O15 Se

Figure S10. ESI(−)-HRMS analysis and UV spectrum of **7.**

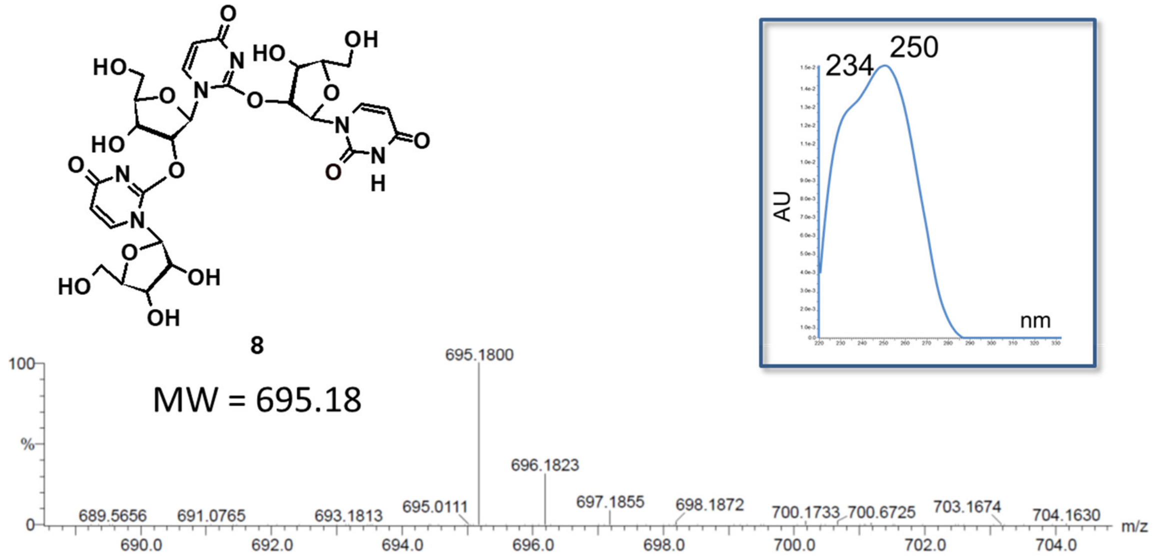

Minimum:				-1.5				
Maximum:		5.0	5.0	80.0				
Mass	Calc. Mass	mDa	PPM	DBE	i-FIT	Norm	Conf(%)	Formula
695.1800	695.1797	0.3	0.4	15.5	311.7	n/a	n/a	C27 H31 N6 O16

Figure S11. ESI(−)-HRMS analysis of and UV spectrum of **8.**

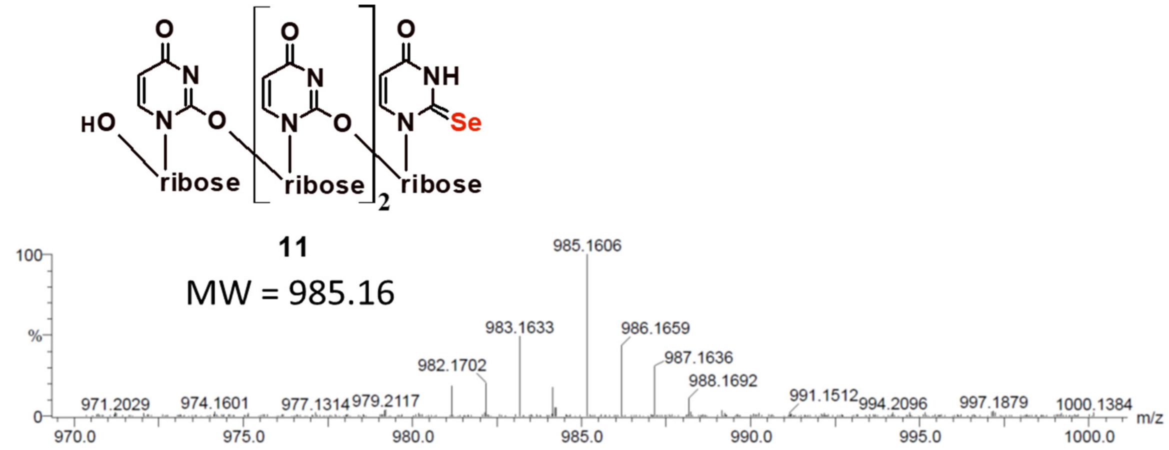

	Mass	Calc. Mass	mDa	PPM	DBE	i-FIT	Norm	Conf(%)	Formula
Minimum:					-1.5				
Maximum:			5.0	5.0	80.0				
	985.1606	985.1602	0.4	0.4	21.5	915.6	n/a	n/a	C36 H41 N8 O20 Se

Figure S12. ESI(−)-HRMS analysis of **11.**

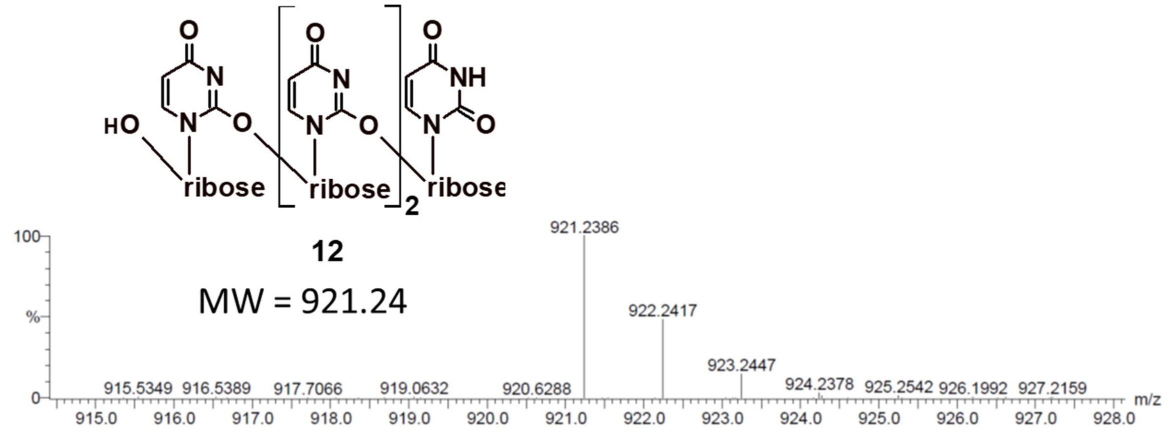

	Mass	Calc. Mass	mDa	PPM	DBE	i-FIT	Norm	Conf(%)	Formula
Minimum:					-1.5				
Maximum:			5.0	5.0	80.0				
	921.2386	921.2386	0.0	0.0	20.5	134.1	n/a	n/a	C36 H41 N8 O21

Figure S13. ESI(−)-HRMS analysis of **12.**

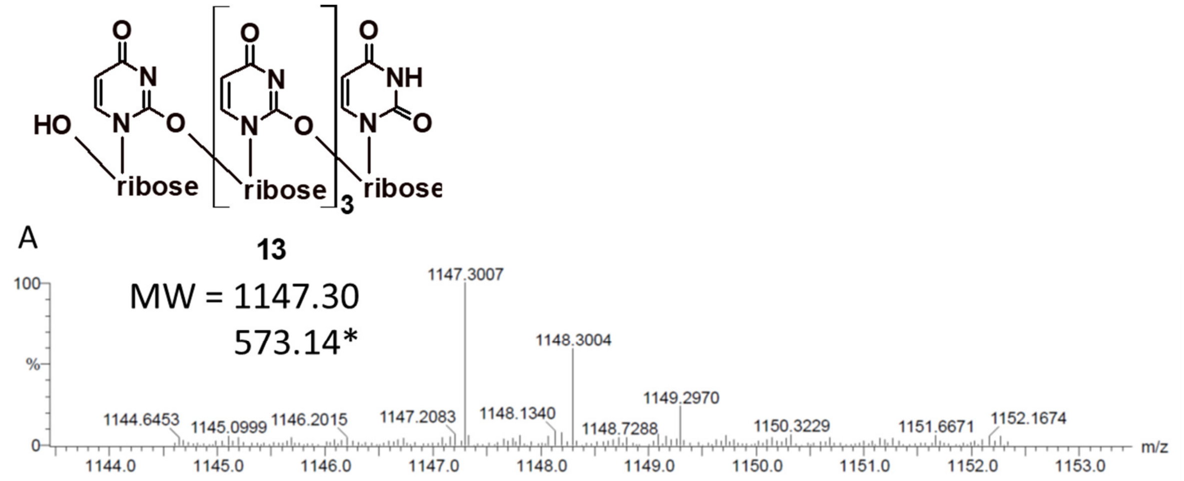

Mass	Calc. Mass	mDa	PPM	DBE	i-FIT	Norm	Conf(%)	Formula
Minimum:				-1.5				
Maximum:		5.0	5.0	80.0				
1147.3007	1147.2976	3.1	2.7	25.5	1151.5	n/a	n/a	C45 H51 N10 O26

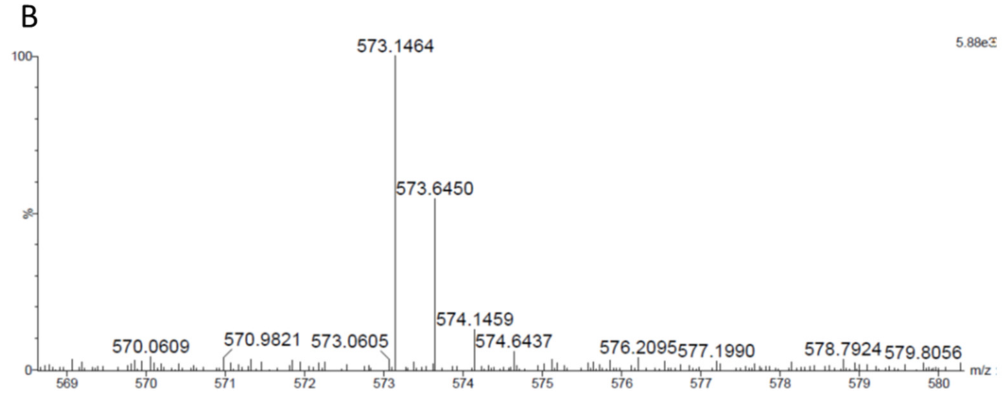

Figure S14. (a) ESI(−)-HRMS analysis of **13;** (b) ESI(-) MS of **13** with peaks correspond the double charged ions.

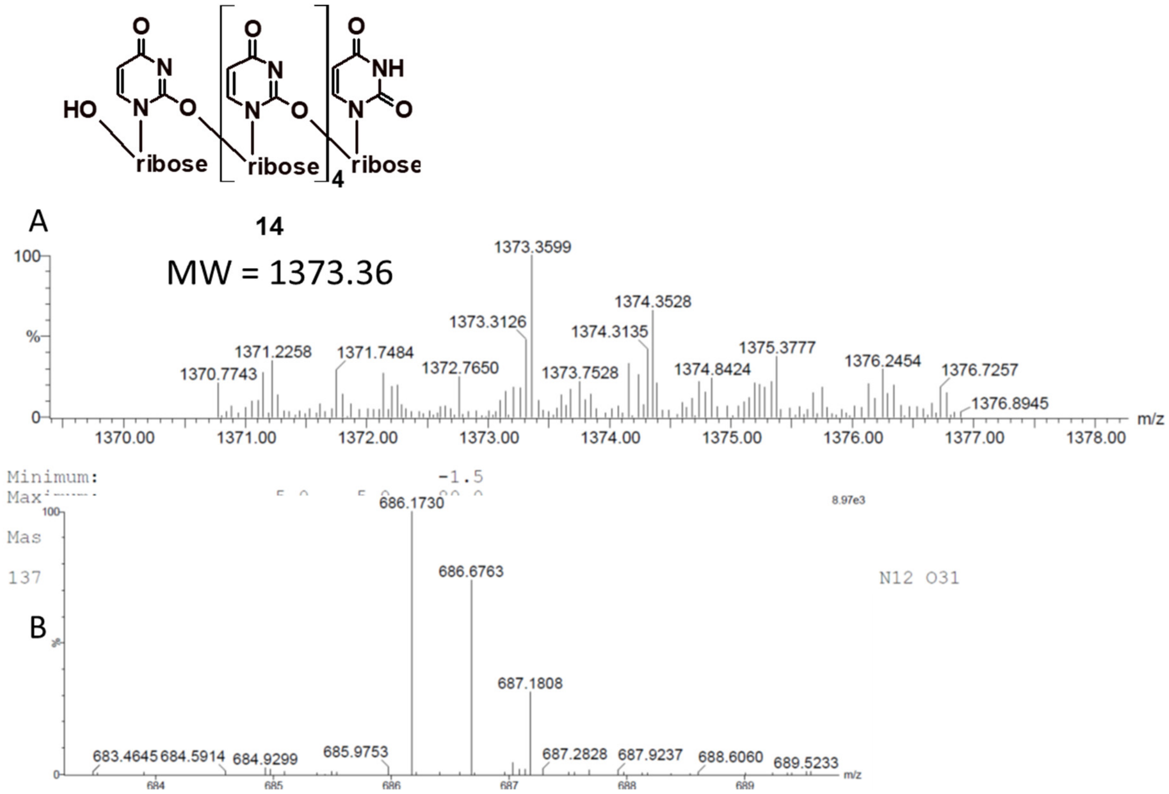

Figure S15. (a) ESI(−)-HRMS analysis of **14**; (b) ESI(-) MS of **14** with peaks correspond the double charged ions.

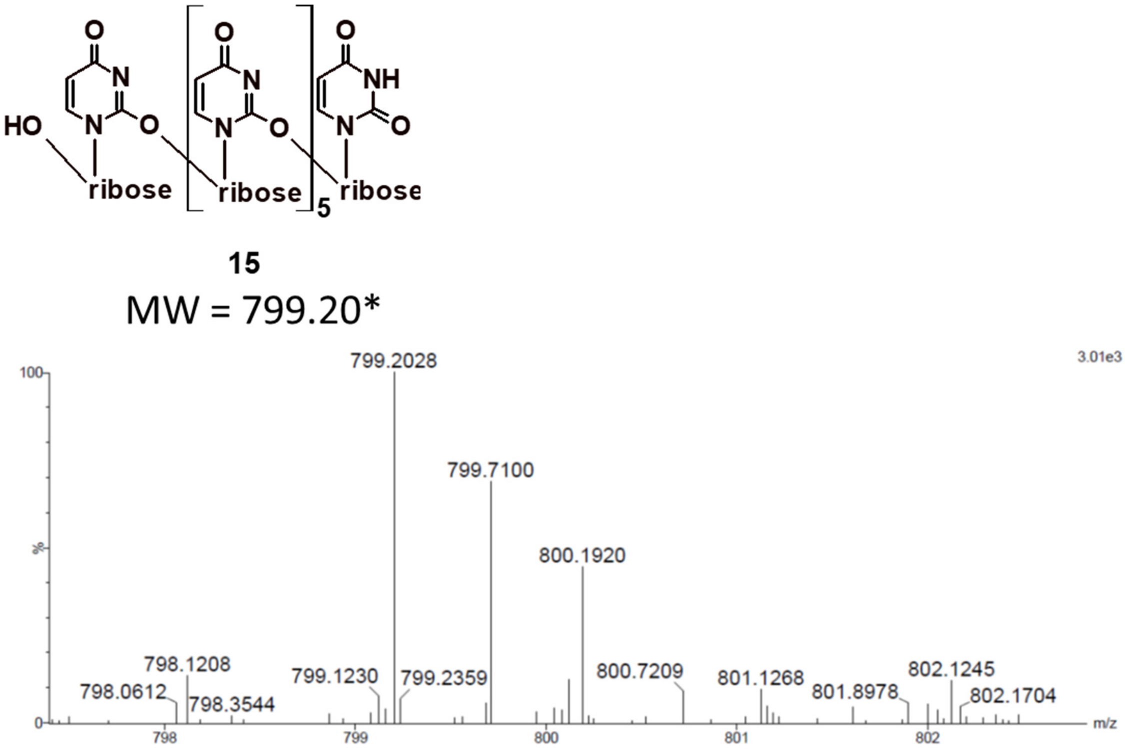

Figure S16. ESI(−)-MS of **15** with peaks correspond the double charged ions.

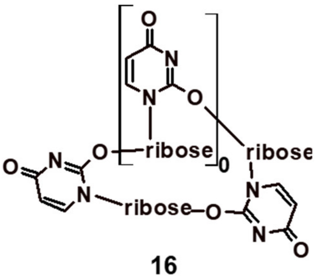

16

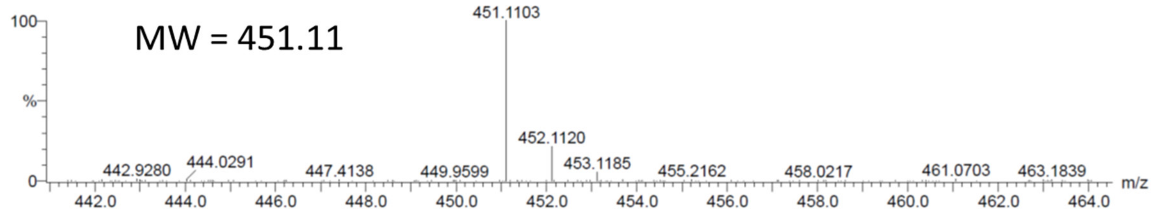

	Mass	Calc. Mass	mDa	PPM	DBE	i-FIT	Norm	Conf(%)	Formula
Minimum:					-1.5				
Maximum:			5.0	5.0	80.0				
	451.1103	451.1101	0.2	0.4	11.5	356.9	n/a	n/a	C18 H19 N4 O10

Figure S17. ESI(−)-HRMS analysis of **16.**

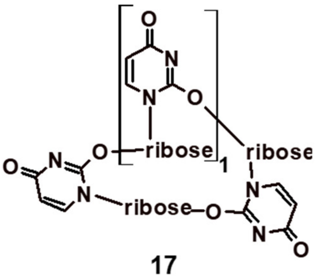

17

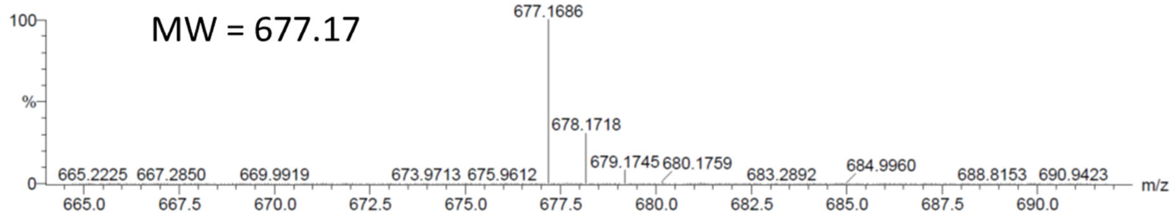

	Mass	Calc. Mass	mDa	PPM	DBE	i-FIT	Norm	Conf(%)	Formula
Minimum:					-1.5				
Maximum:			5.0	5.0	80.0				
	677.1686	677.1691	-0.5	-0.7	16.5	1274.3	0.000	100.00	C27 H29 N6 O15

Figure S18. ESI(−)-HRMS analysis of **17.**

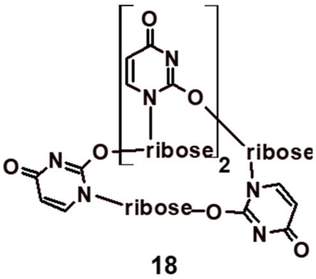

18

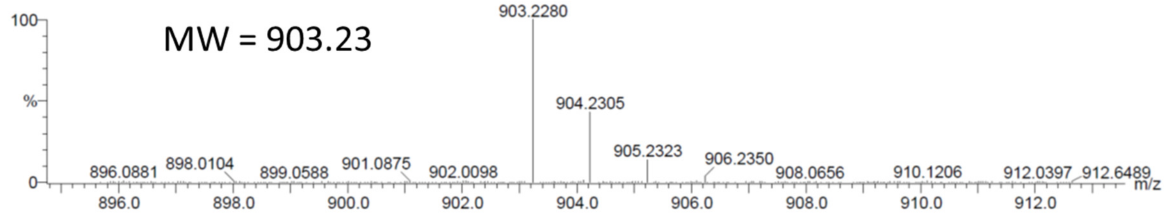

Minimum:				-1.5				
Maximum:		5.0	5.0	80.0				
Mass	Calc. Mass	mDa	PPM	DBE	i-FIT	Norm	Conf(%)	Formula
903.2280	903.2281	-0.1	-0.1	21.5	1237.2	n/a	n/a	C36 H39 N8 O20

Figure S19. ESI(−)-HRMS analysis of **18.**

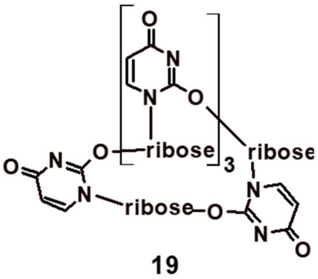

19

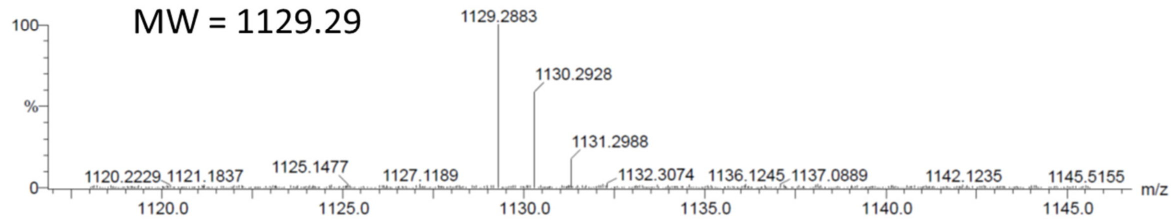

Minimum:				-1.5				
Maximum:		5.0	5.0	80.0				
Mass	Calc. Mass	mDa	PPM	DBE	i-FIT	Norm	Conf(%)	Formula
1129.2883	1129.2870	1.3	1.2	26.5	1345.5	0.271	76.25	C45 H49 N10 O25

Figure S20. ESI(−)-HRMS analysis of **19.**

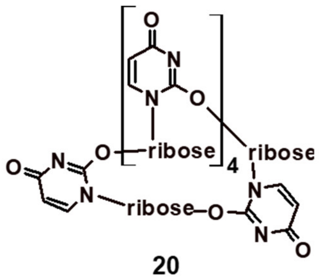

20

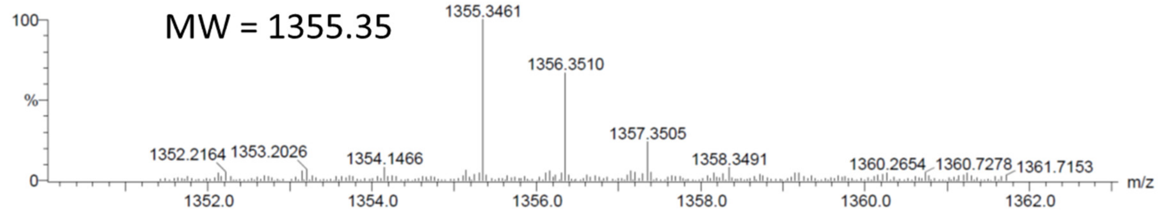

Minimum:				-1.5				
Maximum:		5.0	5.0	80.0				
Mass	Calc. Mass	mDa	PPM	DBE	i-FIT	Norm	Conf(%)	Formula
1355.3461	1355.3460	0.1	0.1	31.5	1731.2	0.242	78.54	C54 H59 N12 O30

Figure S21. ESI(−)-HRMS analysis of **20.**

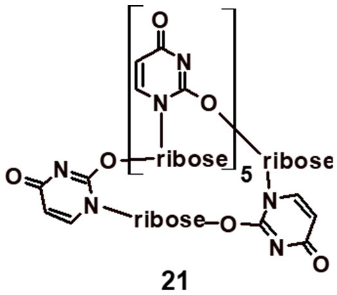

21

MW = 790.20*

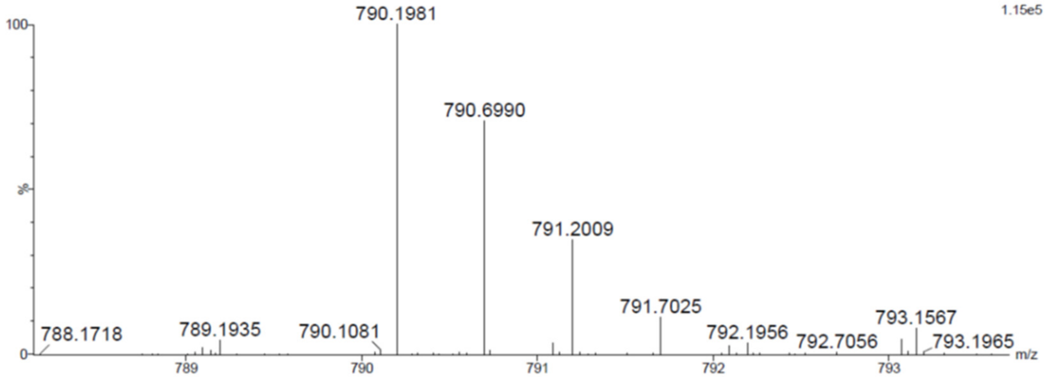

Figure S22. ESI(−)-MS of **21** with peaks correspond the double charged ions.

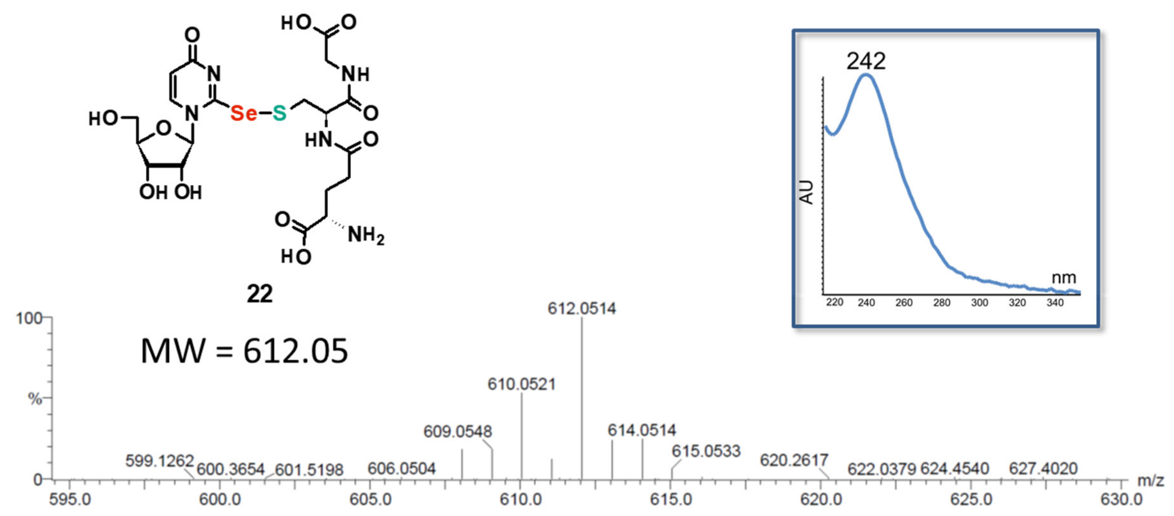

Minimum:				-1.5				
Maximum:		5.0	5.0	90.0				
Mass	Calc. Mass	mDa	PPM	DBE	i-FIT	Norm	Conf(%)	Formula
612.0514	612.0515	-0.1	-0.2	10.5	90.9	0.002	99.82	C19 H26 N5 O11 S Se

Figure S23. ESI(−)-HRMS analysis and UV spectrum of **22.**

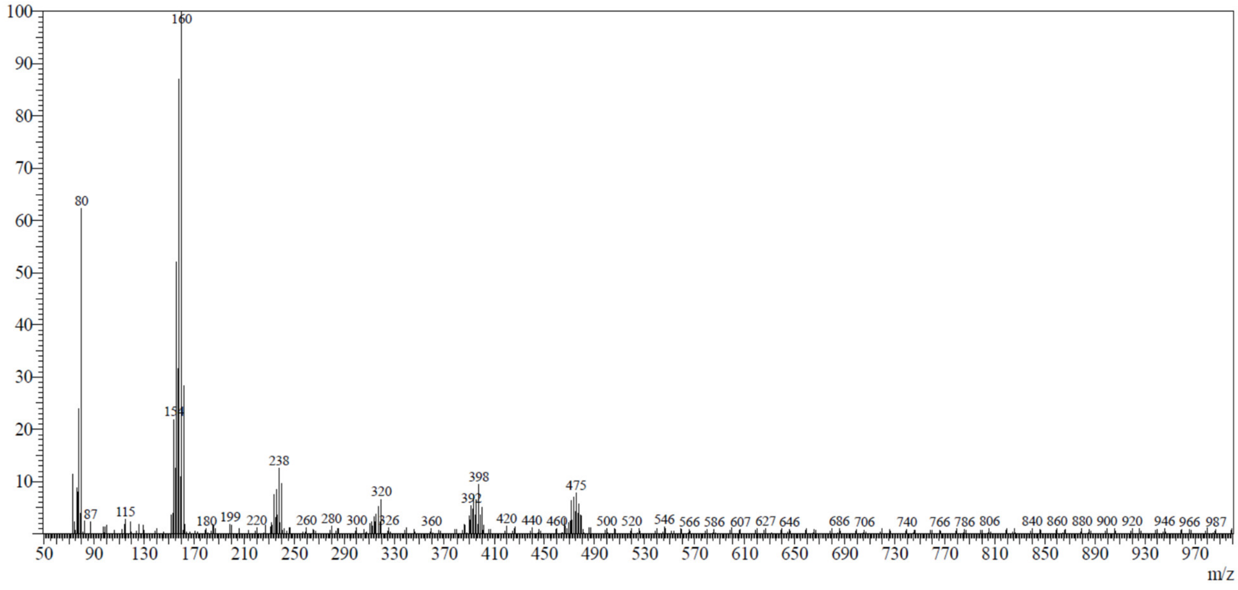

Figure S24. EI (electron impact) mass spectrum of product released as red precipitate.

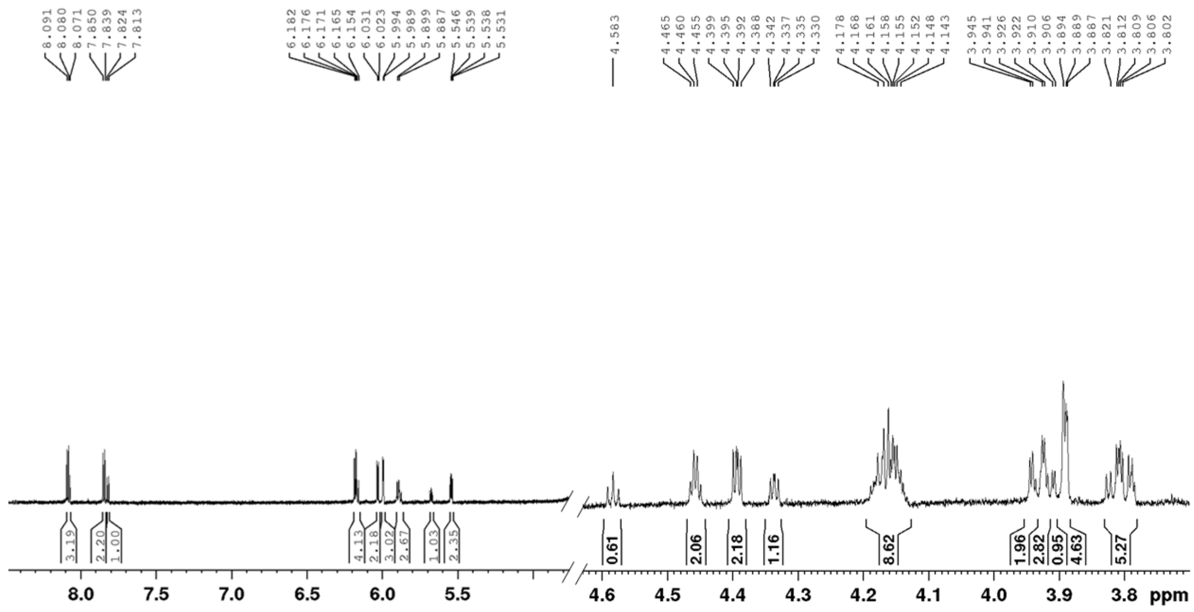

Figure S25. ^1H NMR (700 MHz, D_2O) spectrum of dinucleoside **6.**

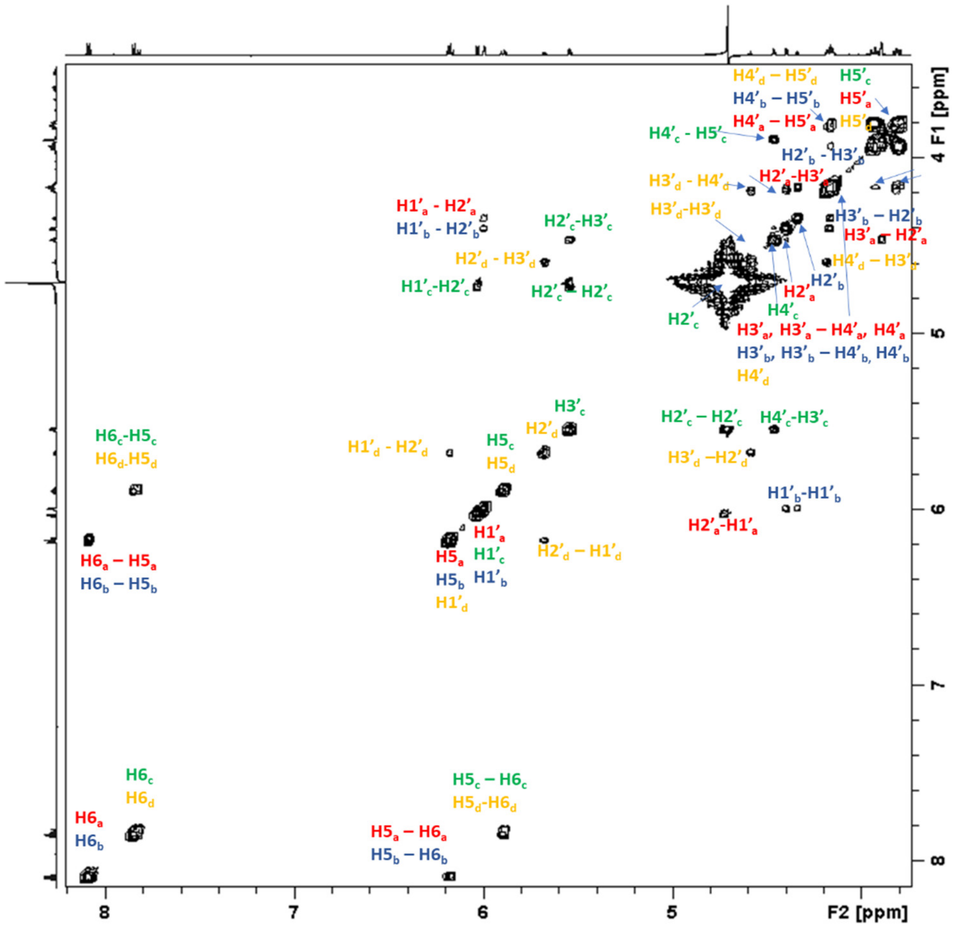

Figure S26. ^1H -^1H COSY (700 MHz, D_2O) of **6.**

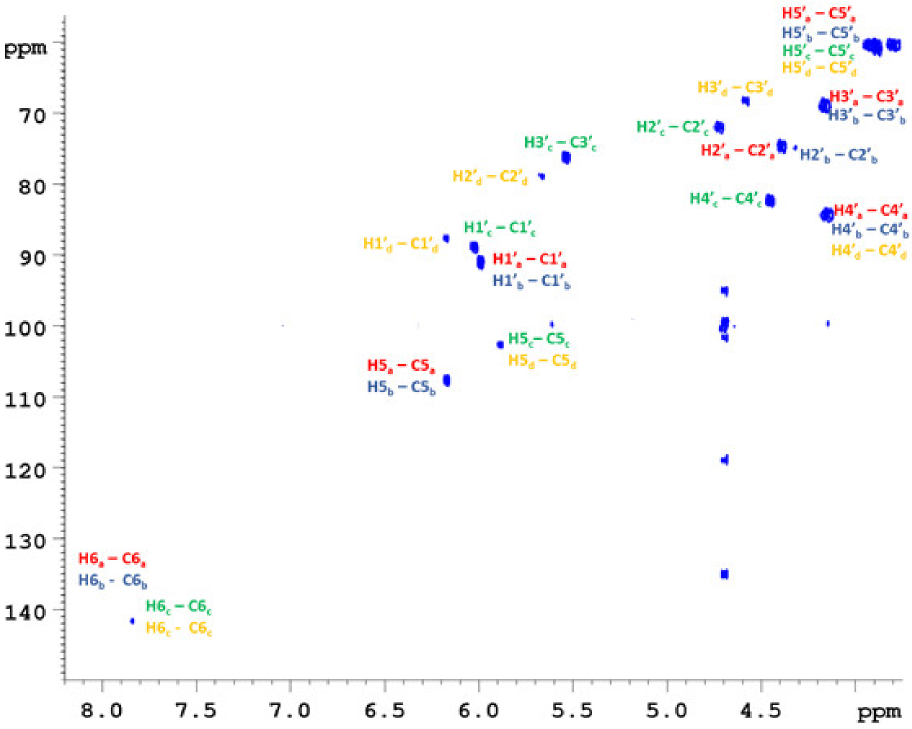

Figure S27. ^{1}H -^{13}C HSQC (700 MHz, D_2O) of **6.**

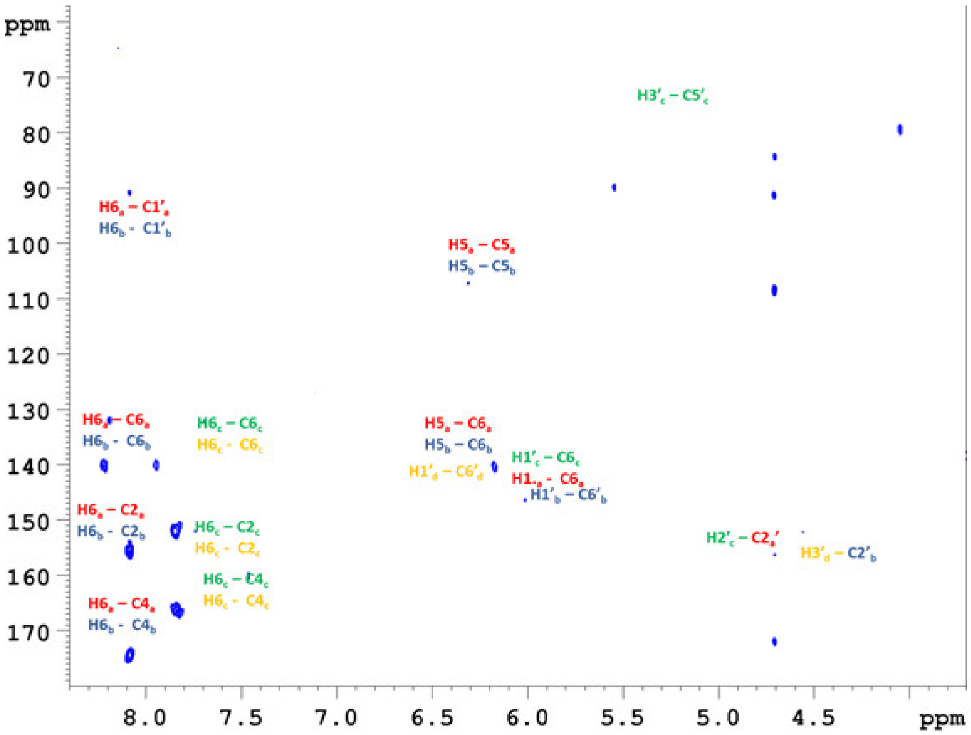

Figure S28. ^{1}H -^{13}C HMBC (700 MHz, D_2O) of **6**. Only the main cross peaks are marked in the spectrum.

Table S1. UPLC-PDA-ESI (-)-HRMS products identified during rescue assay. The UPLC retention time (Rt, min) and *m/z* data (atomic mass unit to its formal charge ratio) for $[M-H]^-$ in negative mode are given.

Compound	UPLC-PDA-ESI(–)-HRMS			
	Elemental Composition	Rt [min]	*m/z* $[M-H]^-$	
			Calcd	Found
DTT	$C_4H_{10}O_2S_2$	3.74	153.0044	153.0048
DTTox	$C_4H_8O_2S_2$	4.01	150.9887	150.9890
Asc	$C_6H_8O_6$	1.09	175.0243	175.0240
Ascox	$C_6H_6O_6$	1.35	173.0860	173.0090
GSH	$C_{10}H_{17}N_3O_6S$	1.15	306.0760	306.0764
GS-SG	$C_{20}H_{32}N_6O_{12}S_2$	0.97	611.1142	611.1440
GS-Se2U	$C_{19}H_{26}N_5O_{11}SSe$	2.33	612.0514	612.0514

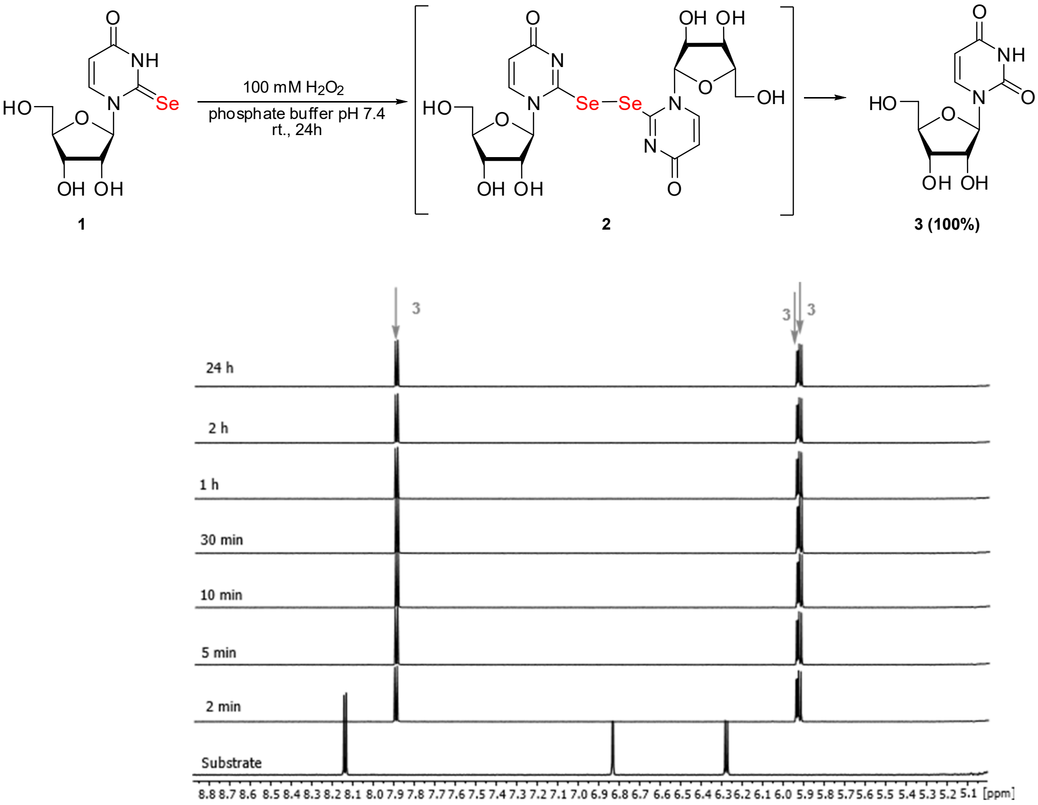

Figure S29. ^1H NMR analysis of the reaction mixtures for oxidation of Se2U (**1**, 10 mM) with H_2O_2 (100 mM) in 67 mM phosphate buffer pH 7.4, at room temperature.

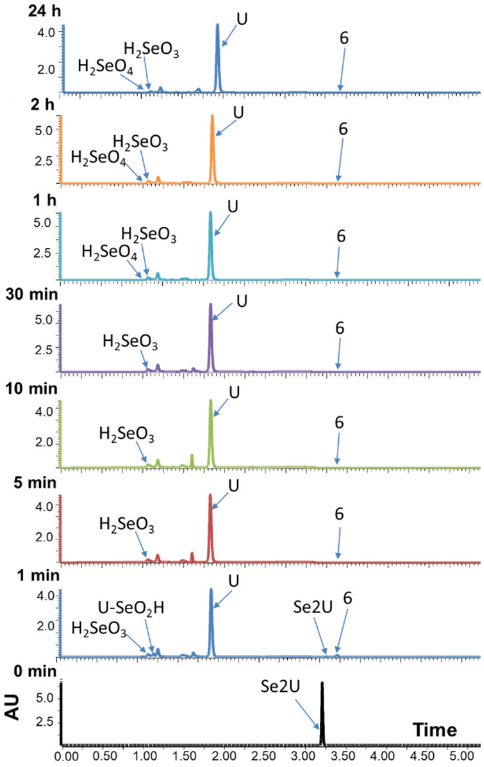

Figure S30. UPLC-PDA chromatographic analysis of the reaction mixtures for oxidation of Se2U (**1**, 10 mM) with H_2O_2 (100 mM) in 67 mM phosphate buffer pH 7.4 , at room temperature. Inorganic selenic acids were identified by UPLC-ESI(−)-HRMS and their retention times were determined based on extracted ion chromatograms (EICs) for the ions corresponding to their deprotonated molecules (*m*/*z* 128.909 and 144.904, respectively).

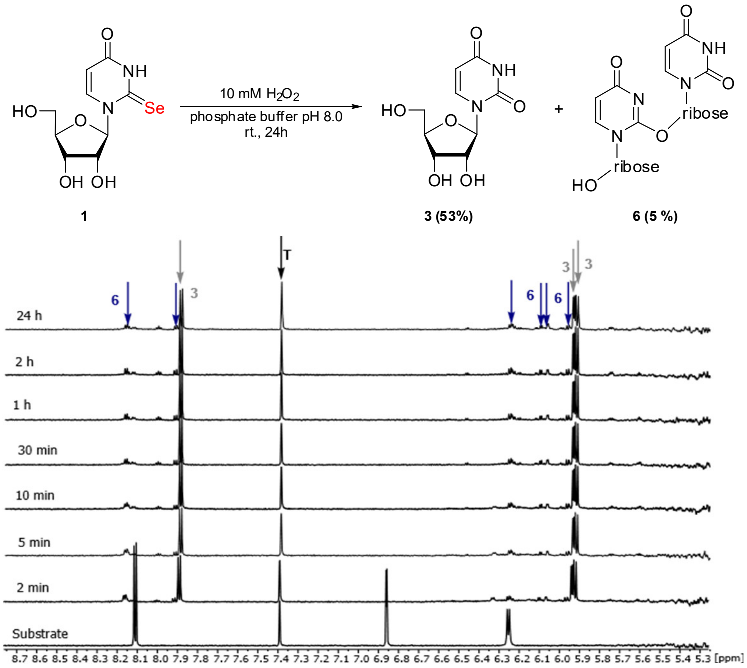

Figure S31. ^{1}H NMR analysis of the reaction mixtures for oxidation of Se2U (**1**, 10 mM) with H_2O_2 (10 mM) in 67 mM phosphate buffer pH 8.0, at room temperature.

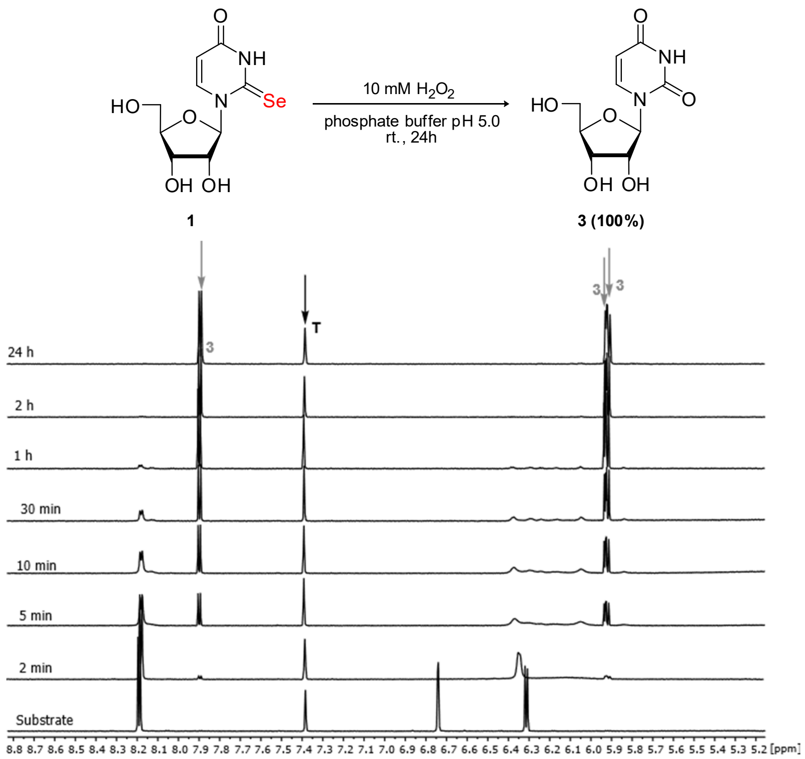

Figure S32. ^{1}H NMR analysis of the reaction mixtures for oxidation of Se2U (**1**, 10 mM) with H_2O_2 (10 mM) in 67 mM phosphate buffer pH 5.0, at room temperature.

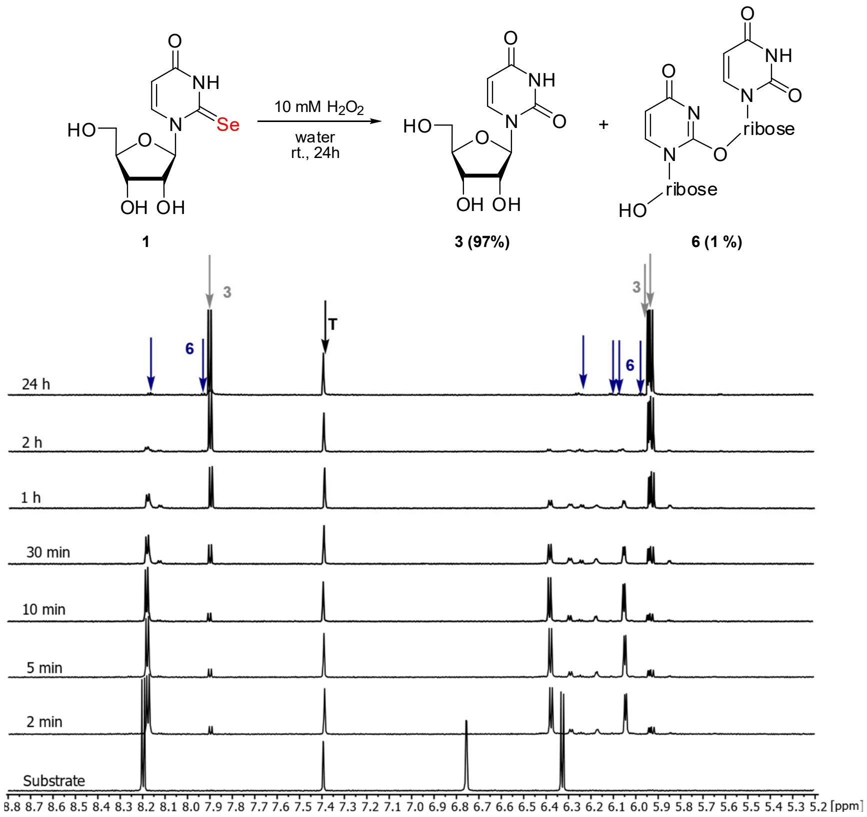

FigureS33. ^{1}H NMR analysis of the reaction mixtures for oxidation of Se2U (**1**, 10 mM) with H_2O_2 (10 mM) in water, at room temperature.

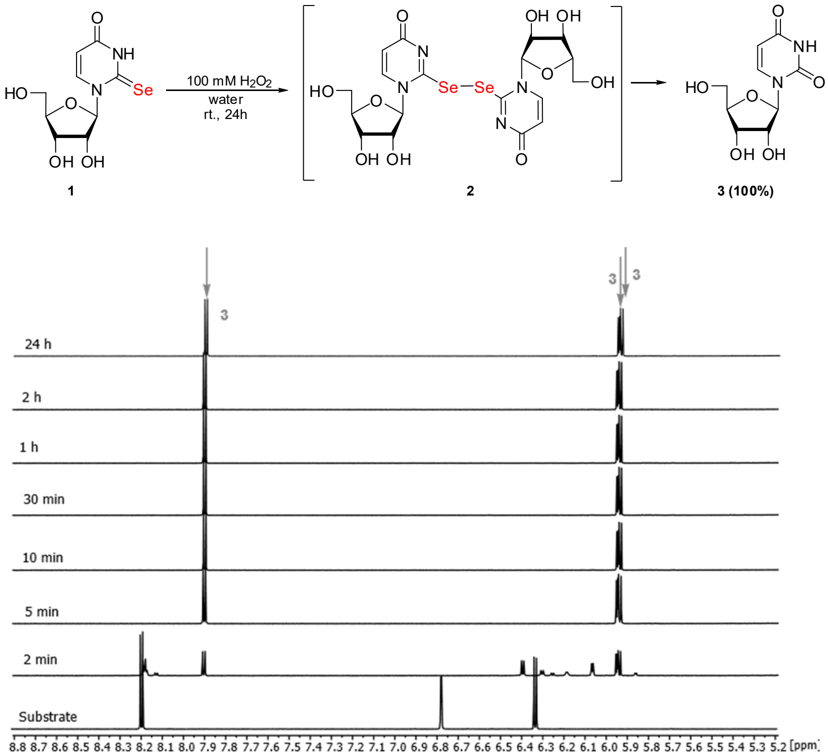

Figure S34. ^{1}H NMR analysis of the reaction mixtures for oxidation of Se2U (**1**, 10 mM) with H_2O_2 (100 mM) in water, at room temperature.

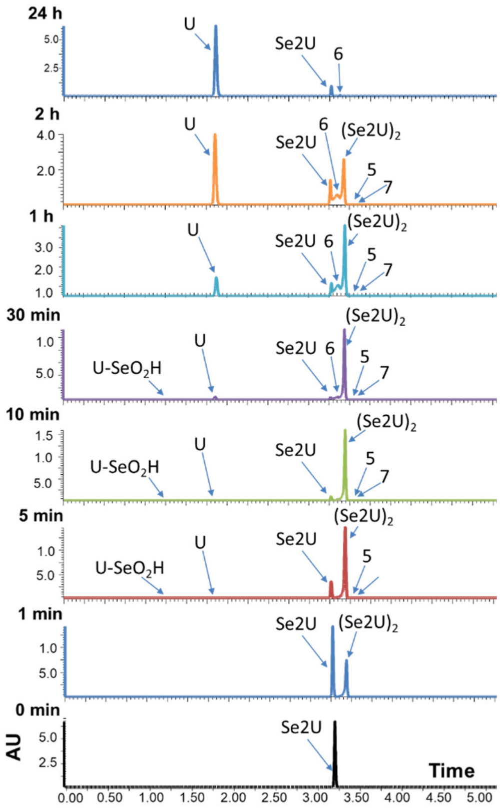

Figure S35. UPLC-PDA chromatographic analysis of the reaction mixtures for oxidation of Se2U (**1**, 10 mM) with H_2O_2 (10 mM) in deionized water, at room temperature.

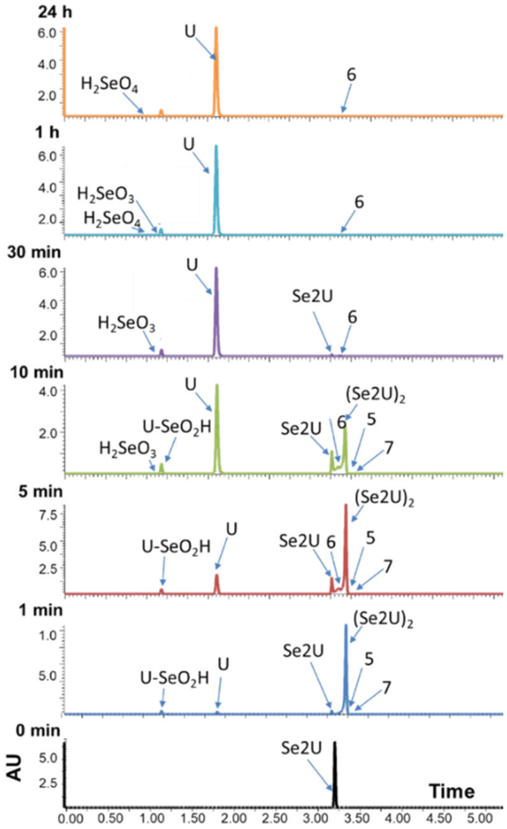

Figure S36. UPLC-PDA chromatographic analysis of the reaction mixtures for oxidation of Se2U (**1**, 10 mM) with H_2O_2 (100 mM) in deionized water, at room temperature.

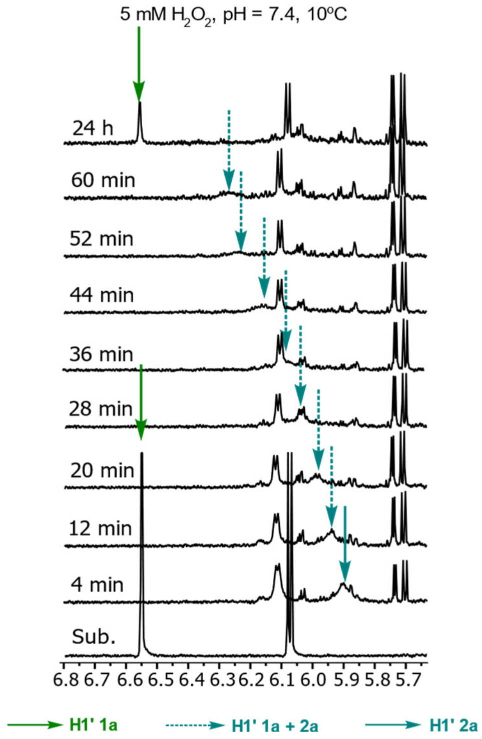

Figure S37. ^{1}H NMR analysis of the reaction mixtures for oxidation of Se2U (**1**, 10 mM) with H_2O_2 (5 mM) in 67 mM phosphate buffer pH 7.4, at 10 °C.

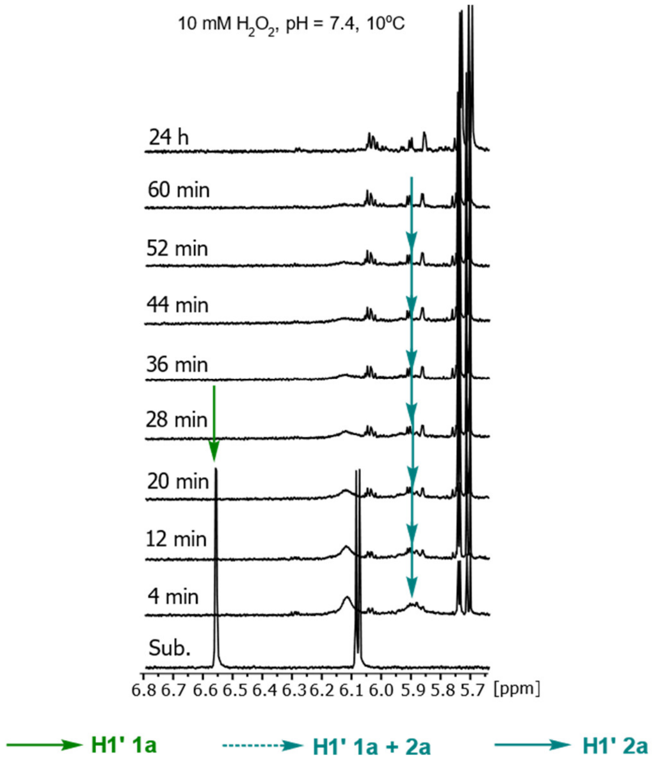

Figure S38. ^{1}H NMR analysis of the reaction mixtures for oxidation of Se2U (**1**, 10 mM) with H_2O_2 (10 mM) in 67 mM phosphate buffer pH 7.4, at 10 °C.

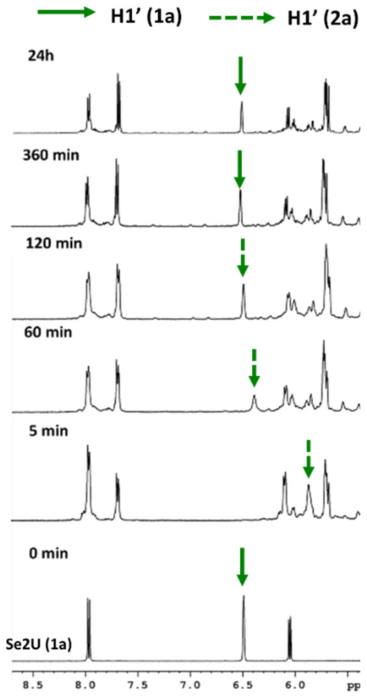

Figure S39 ^{1}H NMR analysis of the reaction mixtures for the oxidation of Se2U (**1**, 40 mM) with H_2O_2 (20 mM) in phosphate buffer pH 7.4 at 10 °C.

References

1. Leszczynska, G.; Cypryk, M.; Gostynski, B.; Sadowska, K.; Herman, P.; Bujacz, G.; Lodyga Chruscinska, E.; Sochacka, E.; Nawrot, B. *Int. J. Mol. Sci.* **2020**, *21*, 2882. https://doi.org/10.3390/ijms21082882.
